# Compromised two-start zigzag chromatin folding in immature mouse retina cells driven by irregularly spaced nucleosomes with short DNA linkers

**DOI:** 10.1093/nar/gkaf457

**Published:** 2025-06-04

**Authors:** Brianna Kable, Stephanie Portillo-Ledesma, Evgenya Y Popova, Nathan Jentink, Matthew Swulius, Zilong Li, Tamar Schlick, Sergei A Grigoryev

**Affiliations:** Department Biochemistry & Molecular Biology, Penn State University College of Medicine, 500 University Drive, Hershey, PA 17033, United States; Department of Chemistry and Simons Center for Computational Physical Chemistry, New York University, New York, NY 10003, United States; Department of Neural and Behavioral Sciences, Penn State University College of Medicine, 500 University Drive, Hershey, PA 17033, United States; Department Biochemistry & Molecular Biology, Penn State University College of Medicine, 500 University Drive, Hershey, PA 17033, United States; Department Biochemistry & Molecular Biology, Penn State University College of Medicine, 500 University Drive, Hershey, PA 17033, United States; Department of Chemistry and Simons Center for Computational Physical Chemistry, New York University, New York, NY 10003, United States; Department of Chemistry and Simons Center for Computational Physical Chemistry, New York University, New York, NY 10003, United States; Courant Institute of Mathematical Sciences, New York University, 251 Mercer Street New York, NY 10012, United States; New York University–East China Normal University Center for Computational Chemistry, New York University Shanghai, Shanghai 200122, China; Department Biochemistry & Molecular Biology, Penn State University College of Medicine, 500 University Drive, Hershey, PA 17033, United States

## Abstract

The formation of condensed heterochromatin is critical for establishing cell-specific transcriptional programs. To reveal structural transitions underlying heterochromatin formation in maturing mouse rod photoreceptors, we apply cryo-electron microscopy (cryo-EM) tomography, AI-assisted denoising, and molecular modeling. We find that chromatin isolated from immature retina cells contains many closely apposed nucleosomes with extremely short or absent nucleosome linkers, which are inconsistent with the typical two-start zigzag chromatin folding. In mature retina cells, the fraction of short-linker nucleosomes is much lower, supporting stronger chromatin compaction. By cryo-EM-assisted nucleosome interaction capture, we observe that chromatin in immature retina is enriched with *i* ± 1 interactions, while chromatin in mature retina contains predominantly *i* ± 2 interactions typical of the two-start zigzag. By mesoscale modeling and computational simulation, we clarify that the unusually short linkers typical of immature retina are sufficient to inhibit the two-start zigzag and chromatin compaction by the interference of very short linkers with linker DNA stems. We propose that this short linker composition renders nucleosome arrays more open in immature retina and that, as the linker DNA length increases in mature retina, chromatin becomes globally condensed via tight zigzag folding. This mechanism may be broadly utilized to introduce higher chromatin folding entropy for epigenomic plasticity.

## Graphical Abstract

**Graphical Abstract:**
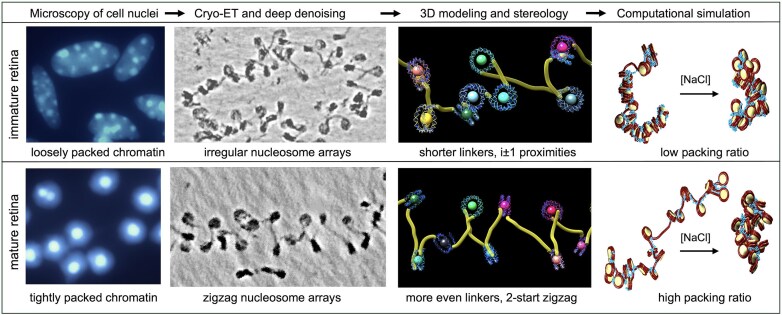

## Introduction

In eukaryotic chromatin, the DNA is discontinuously supercoiled to form “beads-on-a-string” chains of repeated 10-nm units [1–3] called nucleosomes [4]. The nucleosome “beads,” each containing 145–147 bp of DNA making 1.7 left superhelical turns around an octamer of histones H2A, H2B, H3, and H4 in the nucleosome core [5, 6], are connected by variable linker DNA “strings.” The linker DNA length varies dramatically amongst cells from different eukaryotic organisms and tissues [7, 8], ranging from 7 bp in fission yeast [9] to ∼100 bp in echinoderm sperm [10]. The average core and linker DNA lengths combined comprise the nucleosome repeat length (NRL), which can be measured experimentally. Even within the same genome, the NRL in transcriptionally active chromatin domains may be up to 40 bp shorter than that in repressed or noncoding heterochromatin domains [11].

In living interphase cell nuclei and at physiological conditions *in vitro*, nucleosome chains are packed into the higher-order structures regulated by histone modifications, non-histone architectural proteins, and ionic environment [12, 13], reaching about a 400–1000-fold compaction ratio in the interphase cells [14]. Based on earlier EM studies, a hierarchical model was proposed for chromatin folding [15]: the nucleosome “beads-on-a-string” arrays (the primary structural level) first fold longitudinally into the 30-nm chromatin fibers (the secondary structural level) and then self-associate into large chromatin condensates (tertiary structures). The tertiary-level chromatin condensates do not show any distinct secondary structures [16] and acquire a highly dynamic liquid-like behavior [17, 18].

Chromatin 30-nm fiber models have been reconstituted using arrays of regularly spaced nucleosome-positioning sequences [19–21]. Their structural studies by X-ray crystallography and cryo-electron microscopy (cryo-EM) single particle reconstruction showed a prominent two-start zigzag helical organization [22–25]. Extended 30-nm fibers have been observed in the nuclei of some non-dividing cells with condensed chromatin and long NRLs [26, 27]. Capturing nucleosome proximities by three different techniques [28–30], recent cryo-EM imaging [31, 32] and experiments showing that the two-start zigzag topology is recognized by pioneer transcriptional factors capable of inducing cell reprogramming [33] provide evidence for existence and importance of the two-start zigzag *in situ*. However, despite many years of intense study, no extended regularly folded or helical fibers above the 10 nm diameter were observed in the nuclei of proliferating cells and metaphase chromosomes [12, 34–39], suggesting that the two-start topology features do not necessarily reflect a regular 30-nm fiber formation.

Earlier computational modeling [40] and experiments with reconstituted chromatin fibers [41–44] suggested that the chromatin folding irregularity could be caused by the intrinsic variability of the NRL. More recently, mesoscale chromatin models with alternating linker DNA lengths predicted polymorphic chromatin structures [45], and non-uniform linkers were shown to enhance chromatin flexibility and encourage long-range contacts [46]. Still, how variable the individual linkers are in the native chromatin species is largely unknown.

The development of the mammalian retina provides an opportunity to study chromatin structural transitions in the process of cell maturation from proliferating retina progenitors to terminally differentiated non-dividing rod photoreceptors. The retina is a part of the central nervous system and has been studied extensively to analyze epigenetic and chromatin-mediated mechanisms of neuronal differentiation and maturation [47–49] as well as means of reprogramming cell differentiation that could lead to treatment of blindness and improving vision [50–53].

In mouse retina, rod photoreceptors are the largest cell population (∼85%) and can be readily isolated in large numbers for biochemical experiments (>10^6^ cells per retina) [47]. Heterochromatin in rod photoreceptors accumulates in the middle of the nuclei, occupying over 70% of the nuclear diameter, while the remaining euchromatin is located at the nuclear periphery [49, 54]. Though previous works have documented several factors controlling heterochromatin confluence in rod nuclei [53, 55, 56], the molecular and spatial aspects of chromatin higher-order folding remain obscure. In particular, previous *in situ* transmission EM (TEM) imaging of freeze-substituted rod photoreceptors followed by application of Fourier transform suggested the presence of 30-nm structures in the heterochromatin area but no distinct 30-nm chromatin fibers or other regular structures, helical or otherwise [54].

Previously we observed that the dramatic spatial segregation and chromatin condensation during retina development is accompanied by a notable increase in the NRL [48]. Here, to examine the chromatin 3D structural transitions in the process of retina cell maturation, we apply cryo-electron tomography (cryo-ET) that can resolve biological structures embedded in thin layers of vitrified ice at nanoscale resolution [57, 58]. Unlike single-particle reconstruction cryo-EM that relies on averaging images of many thousand identical particles, cryo-ET can resolve individual molecules and molecular assemblies, facilitating analysis of multiplex nucleosome chain conformations in chromatin [59]. Cryo-ET was previously used to image short nucleosome arrays within thin sections of vitrified cell nuclei [31, 32, 60]. With cryo-ET analysis of partially unfolded native nucleosome arrays, we and others revealed a remarkable heterogeneity of linker DNA lengths [61, 62]. Here we report an intriguing relationship between linker length distribution and retina maturation: irregular and extremely short nucleosome spacings in immature retina progenitors compared to wider and more evenly spaced nucleosomes in mature counterparts. By *in situ* formaldehyde crosslinking coupled with cryo-ET, we found that chromatin in immature retina cells is enriched with *i* ± 1 interactions typical of open chromatin in transcribed genes, contrasting the predominately *i* ± 2 interactions in the mature retina, the latter typical of the two-start zig-zag folding. Finally, by computational mesoscale chromatin modeling, we show that the very short nucleosome linkers abundant in the immature retina cells strongly compromise the two-start zigzag formation and chromatin compaction by reducing the potential to form linker DNA stems. We propose that the increase of the nucleosome linker length is a key part of the molecular mechanisms driving heterochromatin spreading and gene repression during retina maturation and that a high frequency of short DNA linkers may be a general way of maintaining a more open euchromatin to ensure high epigenetic plasticity in immature progenitor cells.

## Materials and methods

### Laboratory animals

Wild-type C57Bl/6J (catalog #000664) mice were purchased from the Jackson laboratory and housed in a room with an ambient temperature of 25°C, 30%–70% humidity, a 12 h light–dark cycle, and *ad libitum* access to rodent chow. This study was conducted using both male and female mice in accordance with the National Research Council’s Guide for the Care and Use of Laboratory Animals and all animal experiments were approved by the Penn State University College of Medicine Institutional Animal Care and Use Committee (protocol #00929).

### Retina collection, isolation of retina cell nuclei, and native chromatin

Retinas were dissected from whole eyes at the specified developmental stages as described [48] and placed in phosphate buffered saline (PBS). For *in situ* crosslinking experiments, retinas immersed in PBS were treated with 0.2%–0.4% formaldehyde for 15 min at room temperature with rotation. To stop formaldehyde crosslinking, glycine was added to a concentration of 125 mM and the mixture placed on ice for 5 min. For retina cell disassociation (both native and crosslinked), the retinas were triturated into a cell suspension by repeatedly pipetting up and down with a 1 ml plastic pipet tip, and the cell suspension was centrifuged for 3 min at 50 × *g* (4°C). Cell nuclei were isolated as described [48] and resuspended in RSB buffer (10 mM NaCl, 3 mM MgCl_2_, 10 mM HEPES, pH 7.5), 1 mM phenylmethylsulfonyl fluoride (PMSF), and protease inhibitor cocktail (Sigma P8849).

Isolated nuclei were incubated in RSB with 2 mM CaCl_2_ for 5 min in a 37°C water bath. Micrococcal nuclease (MNase, Roche, #10107921001) was added at 0.0125 μg/ml and incubated for 5 min at 37°C with occasional shaking. To stop MNase digestion, 5 mM Ethylenediaminetetraacetic acid (EDTA, Promega, #4231) was added and placed on ice for 5 min. The nuclei were spun down for 5 min at 11 000 × *g* at 4°C. The pelleted nuclei were resuspended in cold TE buffer (10 mM Tris, 1 mM EDTA, pH 8.0) and incubated overnight at 4°C with rotation. The suspension was centrifuged again for 5 min at 11 000 × *g* at 4°C. The soluble supernatant (S2) was collected. The final nuclear residue (NR) was resuspended in TE buffer. DNA concentration of the S2 and NR chromatin was measured by UV A_260_ spectroscopy. For assessment of DNA size following MNase digestion, aliquots of S2 chromatin were treated with 1% SDS and 0.5 mg/ml proteinase K for 1 h at +55°C and subjected to DNA electrophoresis. For biochemical analysis and imaging, the chromatin samples were dialyzed against HNE buffer (5 mM NaCl, 0.1 mM EDTA, 10 mM HEPES–NaOH, pH 7.5) for 24 h in Slide-A-Lyzer™ MINI Dialysis Devices (Thermo Scientific, #69572).

### Cryo-electron microscopy and tomography

Retina chromatin samples with a concentration of 0.2 mg/ml DNA were mixed with 10 nm fiduciary gold particles (Sigma–Aldrich, cat. #741957) treated by bovine serum albumin, applied to Quantifoil R2/2 200 mesh copper grids (EMS Q250-CR2), and vitrified by plunging into liquid ethane using FEI Vitrobot Mk IV Grid Plunging System as described [62]. Cryo-EM imaging was conducted on Titan Krios G3i 300 kV electron microscope, equipped with a K3 direct electron detector (Gatan, CA) at the Penn State Hershey cryo-EM core as described [62]. Tilt series were aligned using fiducials, CTF corrected, and reconstructed by simultaneous iterative reconstruction technique (SIRT) using IMOD [63] software suite (https://bio3d.colorado.edu/imod/). The chromatin samples, vitrification conditions, raw tilt series, and resulting cryotomograms are listed in Supplementary Table S1.

### Regression tomogram denoising and 3D visualization

Regression denoising was accomplished in Dragonfly (ORS) using data synthesized by cryo-TomoSim as described [62, 64]. Regression-denoised images exported as .tiff files were converted to .mrc files using the *mrc2tif* program within IMOD. Regression-denoised images were segmented into smaller subtomograms by IMOD/3dmod and inverted using “newstack” to generate subtomograms with positive intensity corresponding to high density. Data were visualized either with either IMOD or Chimera.

### Stereological modeling and analysis

Stereological measurements were conducted as described before [62]. Briefly, the SIRT-reconstructed tomograms were segmented into smaller subtomograms by IMOD/3dmod, inverted using “newstack,” and filtered by nonlinear anisotropic diffusion using IMOD command: “nad_eed_3d -n 30 -f -k 50” to reduce noise and enhance chromatin edges. The filtered subtomograms (both SIRT-reconstructed and regression-denoised) were exported into UCSF Chimera [65] (RBVI, Univ. San Francisco, CA), the imaged volumes were fitted with nucleosome core DNA X-ray crystal structure (pdb 2CV5 [66]) semi-automatically using the “fitmap” command, and the nucleosomes were overlaid with centroids, center-to-center axes, and nucleosome planes using the structure analysis “Axes/Planes/Centroids” tool. Each nucleosome in an array was numbered, and the following measurements were recorded: center-to-center distance *D* to the next nucleosome (*n* + 1) in the array; center-to-center distance *N* to the nearest nucleosome (*n*_x_) in the 3D space; angle *α* between the two axes connecting each nucleosome with the previous one (*n*− 1) and the next one (*n* + 1) in a chain: angle *β* between the planes of consecutive nucleosomes *n* and *n* + 1; and angle *para* between the plane of each nucleosome (*n*) and the plane of the nearest nucleosome (*n*_x_) in the 3D space (Supplementary Fig. S1A).

Air–water interfaces were visualized by regression-denoised tomograms as described [62]. All nucleosomes with centers closer than 5 nm to the air/water boundary and that could be damaged by the air contact were excluded from stereological analysis. For a relatively minor fraction of nucleosomes (5.5% of total PN1 nucleosomes and 7.5% of total PN56 nucleosomes vitrified in HNE), the linkers were not resolved due to gaps in EM densities, and corresponding distances *D* and angles *α* and *β* were excluded from the statistical analysis. All distance and angle measurements for individual nucleosome arrays are included in Supplementary Table S2; the data excluded from stereological measurements are highlighted by yellow.

Open linker DNA exiting and entering consecutive fitted nucleosomes were manually traced for arrays containing from 7 to 30 nucleosomes with Chimera Volume Tracer tool using options: “place markers on high density” and “link consecutively selected markers.” Raw multi-segment open linker DNA linker lengths (*O*) were measured by Chimera “measure pathlength sel” command in nanometers and converted to the number of base pairs by assuming 0.34 nm per DNA base pair. The positions at which the open DNA regions were exiting and entering the fitted 146 bp nucleosome cores were indicated as start (s) and end (e) points. Open DNA lengths (*O*) were measured between the start and end points. The DNA segments between the start and end points and the boundaries of the nucleosome core (*S* and *E*, respectively) were subtracted from the nucleosome core DNA length (146 bp), resulting in the constrained core DNA length (*C*). The same segment distances were subtracted from the measured open DNA length (*O*), resulting in the linker DNA length (*L*); see a scheme in Supplementary Fig. S2A. Supplementary Table S3 shows the *O*, *S*, *E*, *C*, and *L* values for all traced nucleosomes. The linker tracing data excluded from statistical analysis due to gaps in EM densities are highlighted by yellow.

The absolute nucleosome proximity values (*i* ± *k*) were obtained by subtracting the number of each nucleosome (*n*) from the number of its nearest nucleosome (*n*_x_) in the 3D space. The nucleosome contacts were defined as those occurring at distance *N* < 11 nm (double nucleosome disk radii) between the centroids as we did before [28]. Nucleosomes involved in “*trans*” contacts were recorded when two nucleosomes from particles *X* and *Y* were in proximity <11 nm, the linker connections were missing, and the unconnected nucleosomes in particles *X* and *Y* were at >28 nm (probability of physical connectivity at such distance is <1% from the analysis).

### Fluorescence microscopy

For fluorescence microscopy, isolated native and formaldehyde-crosslinked retina nuclei were resuspended in TE buffer at ∼1:100 dilution and incubated at 4°C for 4 h—overnight to facilitate nuclei expansion. The nuclear suspension was gently mixed using a wide-pore pipet tip at 1:1 ratio with 0.2 μg/ml Hoechst 33258 in TE. 0.5 ml aliquots of the nuclei stained with Hoechst 33258 were applied onto Poly-l-lysine glass slides (Thermo Fisher, 47100) and incubated for 30 min in a chamber to settle down. The glass-attached nuclei were washed briefly with TE, incubated in TE for 1–2 h. Slides were imaged by fluorescence microscopy using an inverted Nikon Eclipse 2000 microscope and analyzed with NIS-Elements software. No coverslips were used to prevent bursting of unfixed nuclei while taking area measurements. Nuclei area measurements were taken for ∼200 Hoechst-stained cells for each sample. Statistical analysis of nuclear area was performed with Prism v. 10.0.0 (GraphPad) using one-way ANOVA and Kruskal–Wallis test for multiple comparisons.

### NRL analysis by DNA gel electrophoresis

DNA gel electrophoresis in 1.1% agarose gel (Lonza, SeaKem LE Agarose, #50004) and Tris/acetic acid/EDTA buffer (Bio-Rad, cat. #1610743) was conducted as described with constant buffer recirculation [62]. The gels were post-stained with GelRed (Biotium, #41003) and imaged digitally. For analysis of NRL, the relative migration distance (Rf) of the DNA standards and the DNA bands from two independent samples were measured using ImageJ software [67] (National Institutes of Health). A standard curve was generated by plotting the Rf of the DNA standards against the log (molecular weight) of the DNA standards with Excel (Microsoft Excel for Office 365). The DNA length (bp) of each sample was then calculated with the resulting linear equation. To determine the NRL, the DNA length was divided by the number of nucleosomes represented by the corresponding DNA band.

### SDS–PAGE

For verification of histone protein integrity, native chromatin samples were dissolved in SDS-containing loading buffer, and the electrophoresis was carried out in 18% acrylamide gels as described [68]. Gels were post-stained in Brilliant blue R250 (Fisher Biotech, #FL-04-0598) and imaged digitally.

### Mg^2+^-dependent self-association of chromatin

Mg^2+^-dependent chromatin self-association assays were conducted as described [62]. The percentage of DNA in both the supernatant and pellet fractions was entered into Prism version 10.0.0 for Windows (GraphPad Software) to interpolate a standard curve (Sigmoidal, 4PL, *X* is concentration). The resulting IC50 values were averaged to determine the concentration of MgCl_2_ at which 50% of the chromatin sample was precipitated.

For cryo-ET imaging of Mg^2+^-induced condensation, 0.75 or 2.0 mM MgCl_2_ was added to chromatin samples in HNE buffer and the mixture incubated on ice for 20 min prior to sample vitrification.

### Genomic qPCR

Genomic quantitative real-time PCR (qPCR) was performed on CFX Connect Real-Time PCR System (Biorad) with iTaq Universal SYBR Green Supermix (Bio-Rad, #1725121) according to the manufacturer's instructions. Sequences of the DNA oligonucleotide primers are listed in Supplementary Fig. S3H.

### Quantification and statistical analysis

Violin plots were generated using Prism. All other graphs were generated in Excel (Microsoft). Average and standard deviation values were obtained from at least three tomograms and at least two independent biological samples. *P-*values representing probability associated with a Student’s two-sample unequal variance *t*-test with a two-tailed distribution were calculated using Excel (Microsoft). Nonsignificant difference (ns) is shown for *P* > .05. Datasets with nonsignificant difference were additionally examined by nonparametric Kolmogorov–Smirnov test using Prism. In cases where the values are significant by Kolmogorov–Smirnov test but not by standard *t*-test (due to non-Gaussian distribution), the *P-*values resulting from the latter test are indicated by asterisks and are given in the corresponding figure legends. The numbers of nucleosomes (*n*) or nucleosome arrays (*n*′) accounted for in each test are given in the corresponding figure legends.

### Mesoscale modeling of chromatin fibers

To further investigate changes in chromatin architecture during the maturation of retina cells, we apply our chromatin mesoscale model (reviewed in [69, 70]) to simulate chromatin fibers typical of the PN1 and PN56 stages. To model the chromatin fibers, we set linker DNA length distributions obtained from the cryo-EM images of individual chromatin. We choose the two samples that show the largest differences between PN1 and PN56. For each system, PN1 and PN56, we simulate 23-nucleosome arrays. Due to our model resolution of 8.8 bp for linker DNA beads, that translates to linker DNA lengths of 0, 17.65, 26.47, 35.29, 44.12, 52.94, 61.76, 70.59, 79.41, and 114.71 bp. Both the PN1 and PN56 fibers are simulated with saturated linker histone (LH) of one molecule per nucleosome based on experimental measurements for mouse retina [48]. To study the role of salt concentration on fiber architecture, each system is simulated with a NaCl concentration of 5 and 150 mM, with and without the addition of 1 mM Mg^2+^. The effect of 1 mM Mg^2+^ is introduced implicitly by a phenomenological approach [71]. Specifically, we reduce the repulsion among linker DNAs by setting the Debye length in the DNA–DNA electrostatic energy term to *κ* = 2.5 nm and reduce the DNA persistence length from 50 to 30 nm, based to experimental measurements [72, 73].

Our chromatin mesoscale model combines coarse-grained representations of nucleosome cores, histone tails, linker DNA, and LHs within chromatin fiber arrays. To introduce nucleosome cores with 0 bp linker DNA, we connect two nucleosomes by a spring of 3 nm equilibrium length. Overlapping nucleosomes are allowed due to soft excluded volume terms for nucleosome–nucleosome interactions. Further details on model parameters and energy function are described in the Supplementary data.

We simulate 20 copies each for PN1 and PN56 by Monte Carlo sampling, recording for each an ensemble of 2000 configurations. For each ensemble, we calculate the packing ratio as the number of nucleosomes in 11 nm of fiber, the internucleosome interactions, and tail interactions. For a single trajectory of PN1 and PN56, we create fan plots to represent, along each nucleosomal plane, the linker DNA cumulative and average positional distribution across a single trajectory. For single trajectories, we also assess quantitatively the degree of stem formation, as follows. We measure the distances between the average positions of each pair of beads on the two linker DNAs associated with the same core and assume that stem formation requires the distance between two DNA beads to be <2.5 nm. Details of each property calculation are described in the Supplementary data.

## Results

### Chromatin structural transitions during mouse retina maturation are driven by counterions and occur without significant change in the potential for forming tertiary-level chromatin condensates

**Figure 1.**
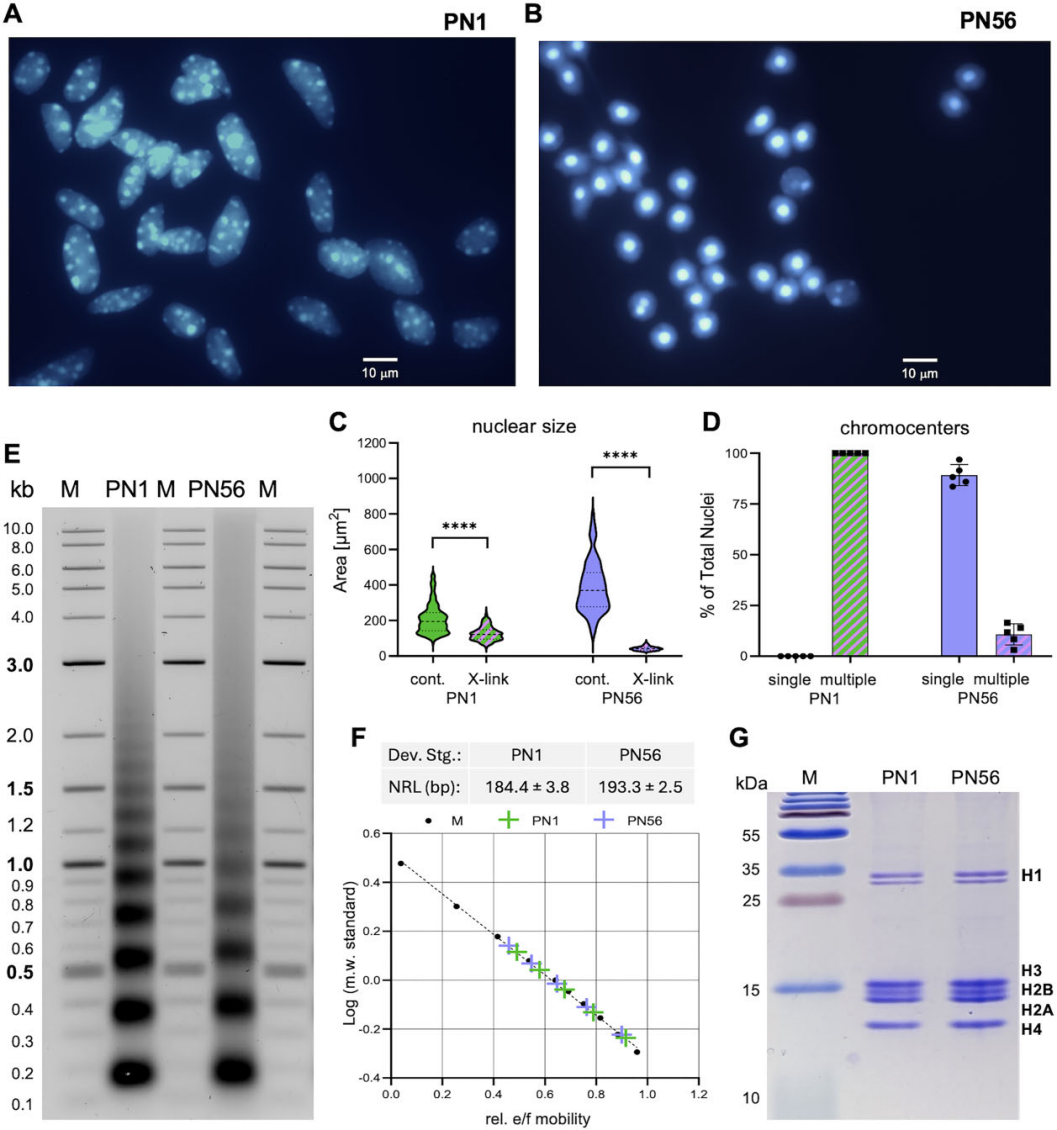
Isolation and characterization of the retina cell nuclei and soluble chromatin. Fluorescence microscopy imaging (Hoechst 33258 staining) of immature PN1 (**A**) and mature PN56 (**B**) nuclei isolated from retina cells crosslinked *in situ* with 0.4% of formaldehyde. (**C**) Violin plots show nuclear area distributions of the intact and crosslinked PN1 and PN56 nuclei. (**D**) Bar graphs show the number of Hoechst-positive chromocenters per nucleus. (**E**) Agarose gel shows DNA size markers (lanes M) and DNA of native MNase-digested chromatin isolated from PN1 and PN56 mouse retina cells. (**F**) Top: NRLs (±SD) determined for PN1 and PN56 mouse retina cells. Bottom: standard curve from the migration distance (Rf) and log(MW) of DNA size standards and NRLs (±SD) determined for PN1 and PN56 mouse retina cells. (**G**) Eighteen percent sodium dodecyl sulfate–polyacrylamide gel electrophoresis (SDS–PAGE) gel stained by Coomassie R250 shows molecular mass markers (lane M) and histones of PN1 and PN56 mouse retina chromatin.

In mature retina rod photoreceptors, the chromatin is largely condensed, and the cell nuclei are much larger than in immature retina [48]. To study chromatin structural transitions underlying retina maturation, we isolated nuclei from immature (PN1, postnatal day 1) and adult (PN56, postnatal day 56) mouse retina. Isolated PN1 nuclei fixed by formaldehyde *in situ* (Fig. 1A) show a uniform morphology typical for eucaryotic cells with multiple foci of constitutive heterochromatin (chromocenters). In comparison, PN56 nuclei (Fig. 1B) have a dramatically reduced size with just one chromocenter, contrasting with multiple chromocenters in PN1 nuclei (Fig. 1C and D), in good agreement with previous *in situ* observations [48, 49, 54]. Only ∼10% of the PN56 nuclei have multiple chromocenters, and larger diameter apparently due to the presence of other retina cells (Fig. 1B and D). This result is consistent with rod photoreceptors being the predominant cell type in mature mouse retina [47, 74] and demonstrates that we can efficiently isolate cell nuclei with either uniformly decondensed (PN1) or predominantly condensed (PN56) chromatin.

In comparison, non-fixed PN56 nuclei expanded in TE buffer appear considerably larger than PN1 nuclei. However, formaldehyde crosslinking *in situ* reverses the trend making PN56 nuclei much smaller than PN1 (Fig. 1C). It thus appears that chromatin in adult retina has a higher potential for salt-dependent contraction than in the immature retina.

We isolated MNase-fragmented soluble chromatin from PN1 and PN56 cells to result in nucleosome arrays containing ∼12 nucleosomes per particle, similar to the size of the nucleosome arrays used in most cryo-EM studies of reconstituted chromatin [19, 23, 24] and our recent study of human chromatin [62]. By DNA electrophoresis, we observed a significant increase in the NRL from ∼184 in PN1 to ∼193 bp in PN56 (Fig. 1E and F). By SDS–PAGE, we observed that both the PN1 and PN56 isolated chromatin contained mostly histones with very little nonhistone protein (Fig. 1G), suggesting that the NRL may be a key variable affecting chromatin folding during retina maturation.

**Figure 2.**
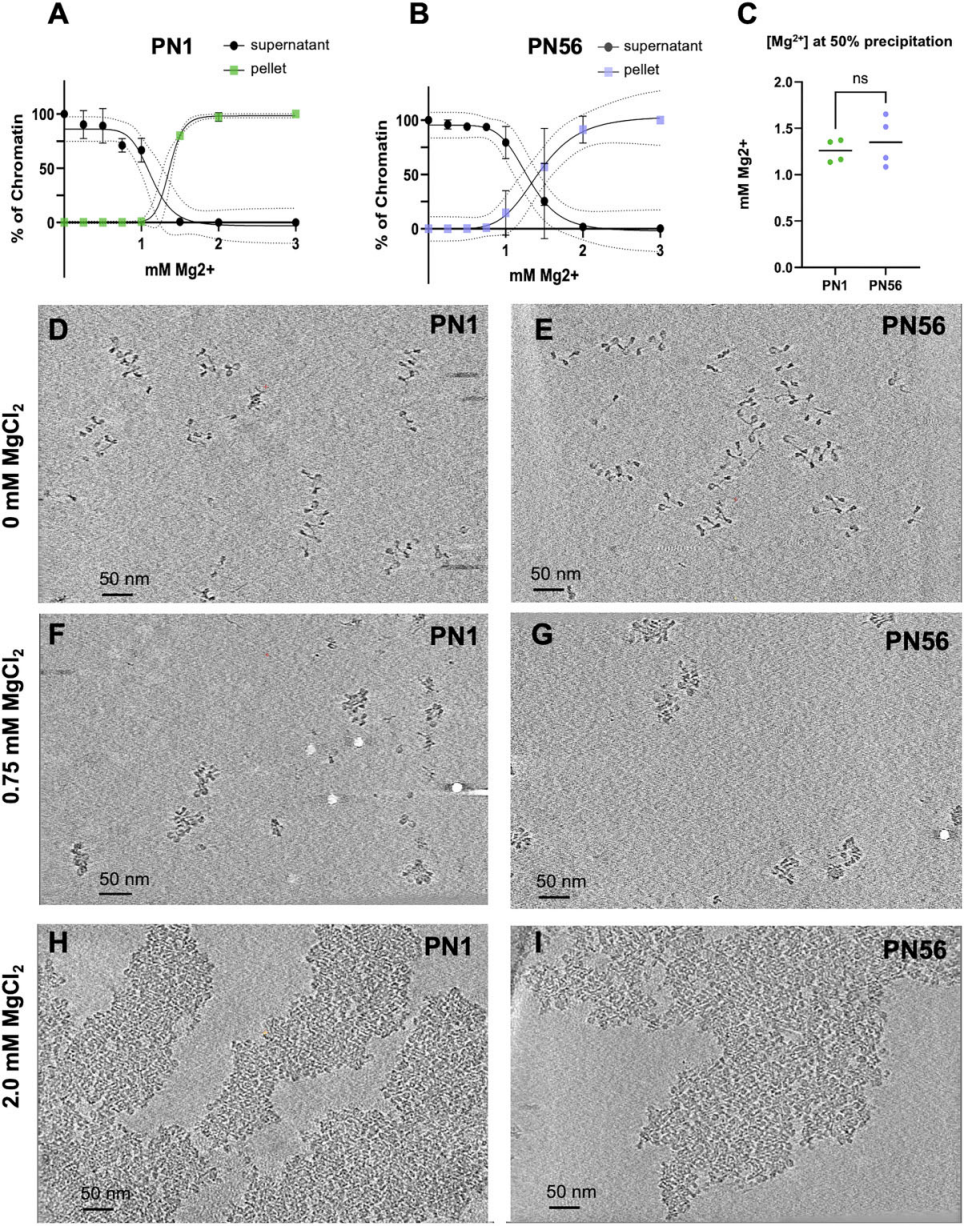
Cryo-ET imaging reveals tight nucleosome packing in Mg^2+^-condensed mouse retina chromatin. (**A**, **B**) Graphs showing the percent ratio of supernatant and precipitated PN1 and PN56 chromatin collected after mixing with the indicated concentrations of MgCl_2_. (**C**) Mg^2+^-induced 50% precipitation points determined for the nucleosome arrays isolated from PN1 and PN56 cells. Representative Z-series slices from cryo-ET tomograms of PN1 (**D**, **F**, **H**) and PN56 (**E**, **G**, **I**) chromatin embedded in vitrified ice at the indicated concentration of Mg^2+^ together with 10 nm fiduciary gold particles show open nucleosome arrays in HNE buffer without Mg^2+^ (D, E), compaction of individual particles at 0.75 mM Mg^2+^ (F, G), and formation of bulky self-associates at 2.0 mM Mg^2+^(H, I). Mag: 53 000×; scale bar 50 nm. Corresponding raw data files with cryo-EM tilt series: D: TS_1_3; E: TS_9_2; F: TS_3_2; G: TS_11_1; H: TS_5_1; and I: TSS_13_1 (Supplementary Table S1).

Our initial hypothesis was that the difference in PN1 and PN56 chromatin condensation is due to an increased potential for cation-driven chromatin self-association in PN56. Therefore, we induced chromatin condensation by divalent cation, Mg^2+^that promotes compaction of nucleosome arrays into zigzag fibers [21, 24] at 0.5–1 mM, which is close to the physiological range of free Mg^2+^ concentration [75]. At concentrations above 1 mM, Mg^2+^ induces the formation of large chromatin condensates [16]. Accordingly, chromatin isolated from both PN1 and PN56 retina cells showed a sharp self-association around 1.1 mM without a significant difference between the two chromatin samples (Fig. 2A–C) and rather similar to the Mg^2+^-dependent self-association of reconstituted nucleosome arrays [16, 76], suggesting that the potential for forming tertiary chromatin condensates is not significantly changed during retina maturation.

We then used cryo-ET to examine native PN1 and PN56 chromatin vitrified in the low-salt HNE buffer with and without added Mg^2+^. With both PN1 and PN56 chromatin in the low-salt HNE buffer, we observed unfolded nucleosome arrays (Fig. 2D and E) very similar to our previous cryo-ET of human chromatin [62]. In the presence of 0.75 mM Mg^2+^, the chromatin showed close juxtaposition of nucleosome disks without any obvious zigzag fibers or other regular structures (Fig. 2F and G). PN1 and PN56 chromatin vitrified at 2 mM Mg^2+^ showed prominent self-association in which nucleosomes merged to form bulky tertiary structures (Fig. 2H and I). While we could not resolve separate nucleosome arrays within the tertiary structures, the nucleosome packing showed apparent stacks of several nucleosomes (seen as edge-to-edge) mostly at the periphery of the chromatin condensates. However, neither the biochemical characterization nor the overall “eagle-eye” cryo-ET visualization of native retina chromatin shows any large-scale structural changes in the process of retina maturation, thus necessitating a more detailed ultrastructural analysis.

### Internucleosome distances increase significantly during retina maturation

**Figure 3.**
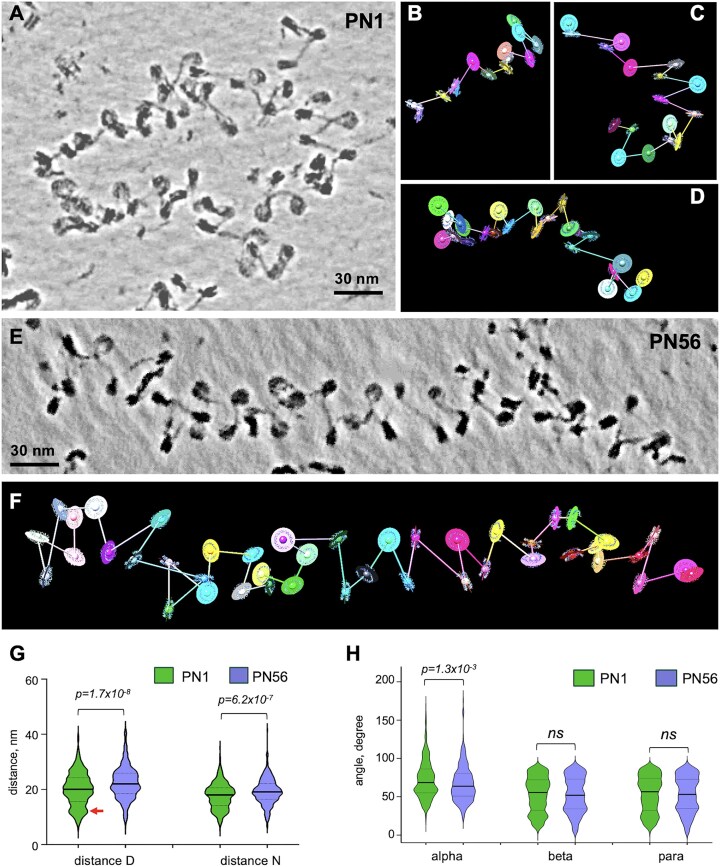
Cryo-ET and stereological modeling reveal significant changes of internucleosome spacing during retina maturation. (**A**) Cryo-ET tilt series of the PN1 chromatin vitrified in HNE buffer (TS_1_1) were tomographically reconstructed and processed by deep learning denoising. A cropped image is shown as a composite of Z-series slices in IMOD. (**B**–**D**) The cropped image of PN1 chromatin shown in panel (A) was processed to generate centroid/axes/plane (CAP) models: B: CAP_1_5; C: CAP_1_3; and D: CAP 1_4. (**E**) Cryo-ET tilt series of the PN56 chromatin in HNE buffer (TS_9_1) were tomographically reconstructed and processed by deep learning denoising. A cropped image is shown as a composite of Z-series slices in IMOD. (**F**) The cropped image of PN56 chromatin shown in panel (E) was processed to generate CAP model (CAP 9_2). Violin plots of distances *D* and *N* (**G**) and angles *α*, *β*, and *para* (**H**) obtained for arrays of PN1 nucleosomes vitrified in HNE buffer [green shapes, *n* = 529 (*D*), 570 (*N*), 468 (*α*), 530 (*β*), and 570 (*para*)] and arrays of PN56 nucleosomes vitrified in HNE buffer [violet shapes, *n* = 344 (*D*), 405 (*N*), 294 (*α*), 347 (*β*), and 380 (*para*)]. See Supplementary Fig. S1A for a scheme of stereological measurements.

To aid 3D visualization and resolve individual nucleosomes, we processed the tomograms of retina chromatin vitrified in HNE (Fig. 2D and E) using deep learning-based regression denoising models (Fig. 3A and E). When comparing the denoised PN1 and PN56 images (cf. Fig. 3A and E), two features become readily apparent. First, PN1 contains nucleosome arrays with less even spacings than those in PN56. Second, PN1 arrays tend to easily change direction and shape in the 3D space, while PN56 arrays tend to have more even zigzag organization and straighter array axis.

For quantitative stereological analysis, we generated CAP models for each nucleosomal array (Fig. 3B–D and F). For each nucleosome in the CAP model, we recorded the center-to-center distance *D*, the center-to-center distance *N*, the angle α between the two axes connecting three consecutive nucleosomes, the angle *β* between the planes of consecutive nucleosome pairs, and the angle *para* between the planes of proximal nucleosomes (schematized on Supplementary Fig. S1A). In addition, we also built CAP models and conducted the same stereological measurements with the corresponding deep learning-denoised cryotomograms, showing an excellent correspondence between the nonlinear anisotropic diffusion filtering and deep learning regression denoising (Supplementary Fig. S1B and C).

Due to the missing wedge and the noisy nature of cryo-ET data, a minority of nucleosomes showed a gap in linker electron density so that 5.4% of all distances *D* in PN1 and 9.2% of all distances *D* in PN56 and corresponding angles *α* and *β* were not accounted for in the modeling (highlighted by yellow in Supplementary Table S2). To test whether the nucleosomes with gaps in electron density might be structurally different from the total nucleosomes, we compared the total internucleosomal distances *N* and angles *para* (both recorded universally for all nucleosomes) with the fraction of those parameters connected without gaps (*D*^+^) and found no significant differences (Supplementary Fig. S1D–G), indicating an absence of nonrandom bias associated with the missing linkers.

Consistent with our MNase analysis showing ∼10 bp increase in the linear length (Fig. 1F), the average distance *D* from one to the next nucleosome was significantly (*P*= 1.7 × 10^−8^) increased from 20.0 to 22.3 nm during retina maturation (Fig. 3G). Remarkably, in comparison to SD = ±3 bp in the MNase-derived NRL, the distribution of individual internucleosome distances was SD = ±5.9 nm in both PN1 and PN56, corresponding to ±17.4 bp (∼0.34 nm per DNA base pair). This high nucleosome spacing heterogeneity is consistent with those previously observed in the metaphase HeLa chromosomes [61] and interphase K562 chromatin [62]. Thus, the high heterogeneity in the nucleosome spacing is likely a general feature common for primary vertebrate tissues (such as mouse retina) and proliferating cell cultures. Another notable feature distinguishing the two samples is the peak of very short nucleosome distances *D* at ∼11.5 nm in PN1 (red arrow in Fig. 3G) absent from PN56. The distributions of nearest neighbor distances *N* (which are not expected to be very different from *D* in the open chromatin) show the same tendencies with a significantly increased (*P*= 6.2 × 10^−7^) *N* values in PN56 and the prominence of 11.5 nm nucleosome distance in PN1 (Fig. 3G). In comparison to PN1 and PN56, for postnatal day 21 (PN21), we observed intermediate *D* and *N* values, confirming that internucleosome distances gradually change during retina development (Supplementary Fig. S1H and I).

In contrast to the significant changes in internucleosomal distances, changes in angles *α*, *β*, and *para* are rather modest (Fig. 3H). Only angle *α* showed a significant (*P*= 1.3 × 10^−3^) decrease from 72.9° to 67.1°; this change reflects an increase in LH H1 during retina maturation [48], which makes the angle between DNA linkers entering and exiting the nucleosome narrower and promotes formation of linker DNA stem [19]. Thus, our stereological analysis strongly indicates that internucleosomal distances reflecting changes in nucleosome linker lengths rather than planar angles reflecting rotational changes between nucleosome orientations are most strongly affected during retina maturation.

### Nucleosome linker length distribution is widely varied in immature retina chromatin

**Figure 4.**
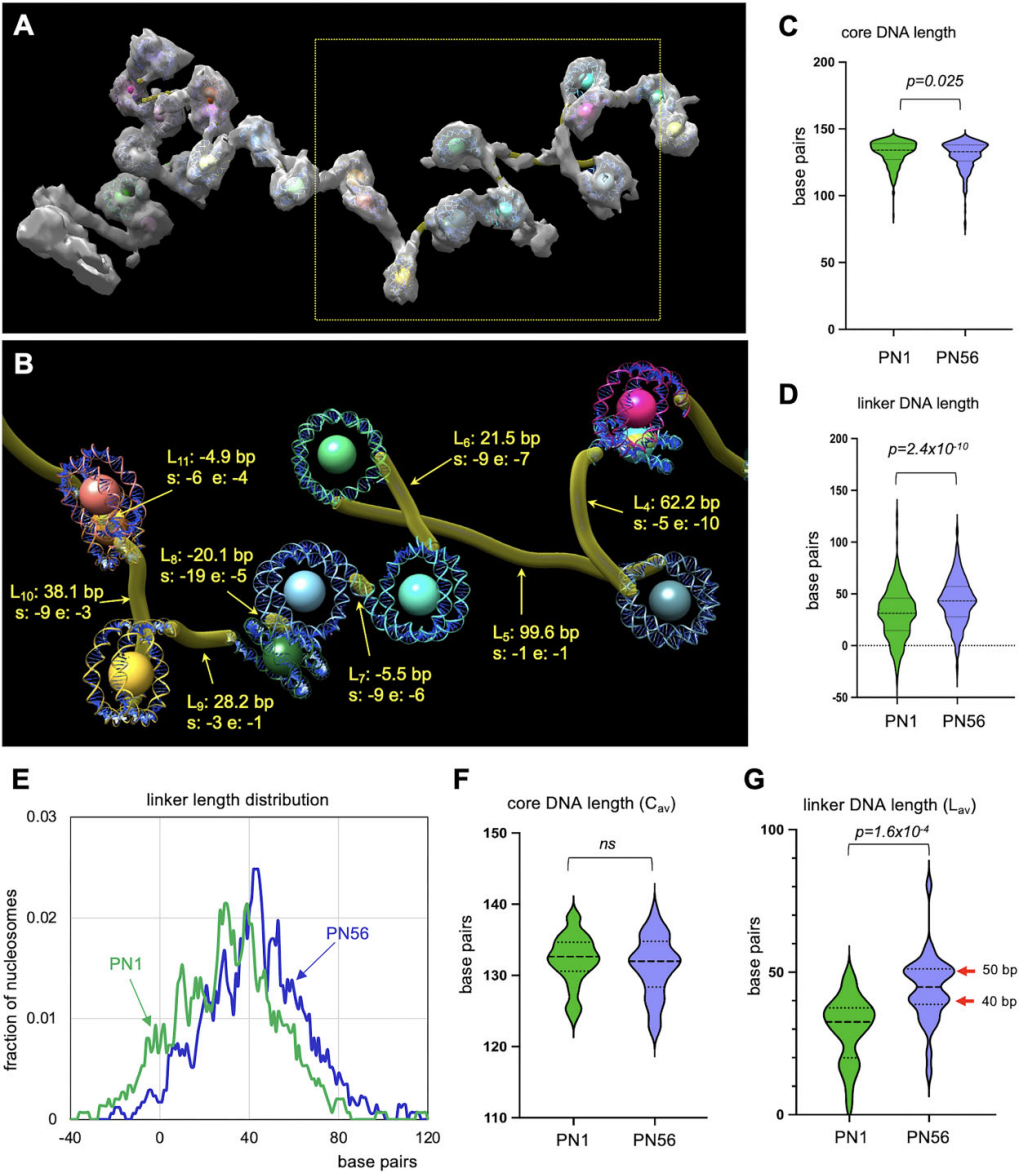
Tracing and comparison of linker DNA length in the PN1 and PN56 chromatin. (**A**) A cryotomogram of the PN1 chromatin vitrified in HNE buffer (TS_1_1) was processed by deep learning denoising, transferred to Chimera to generate CAP model (CAP_1_4), volume-fitted with nucleosome core structures (pdb 2CV5), and shown as an isosurface. (**B**) Open linker DNA in CAP_1_4 manually traced with Chimera Volume Tracer tool. L4 (linker 3) to L11 (linker 11), indicated by arrows, show the measured linker lengths (*L*) and the positions of the open DNA start (s) and end (e) in DNA base pairs. See Supplementary Fig. S2A for a detailed scheme. (**C**) Violin plots show distributions of unpeeled core DNA lengths calculated for PN1 (green, *n* = 300) and PN56 (*n* = 371) nucleosomes as indicated. (**D**) Violin plots show distributions of linker DNA lengths *L* calculated for PN1 (*n* = 298) and PN56 (*n* = 378) nucleosomes as indicated. (**E**) Frequency distribution profile of linker length *L* calculated for PN1 and PN56 nucleosomes as indicated by arrows. (**F**) Violin plots show distributions of average core DNA lengths (*C*_av_) per array for PN1 (*n* = 27) and PN56 (*n* = 32) as indicated. (**G**) Violin plots show distributions of average linker DNA lengths (*L*_av_) per array for PN1 (*n* = 27) and PN56 (*n* = 32) as indicated.

Our stereological analysis accurately measures the internucleosome distances and angles but does not allow one to measure the length of individual linkers that may be differentially folded or peeled off the nucleosome and thus conceal the actual length of DNA connecting the nucleosomes. To further investigate the mechanism generating the wide variation of internucleosome distances, we estimated the DNA linker length distribution by fitting nucleosome cores into denoised images of open nucleosome arrays (Fig. 4A), manually tracing the open DNA regions using Chimera Volume Tracer tool (Fig. 4B), and subtracting the unwrapped nucleosome core DNA segments (see scheme in Supplementary Fig. S2A), resulting in the measured constrained core DNA length *C* and linker DNA length *L* values (listed in Supplementary Table S3).

Comparison of average core DNA length between PN1 and PN56 (Fig. 4C) shows only a minor difference (132.0 bp in PN1 versus 130.2 bp in PN56) that cannot explain the observed difference in nucleosome spacing. Concurrently, average linker DNA length is increased from 30.7 bp in PN1 to 42.6 bp in PN56 (Fig. 4D), showing that the increased nucleosome spacing is mostly due to the increased linker DNA length. When added to the number of the base pairs in the fitted nucleosome core PDB2CV5 (146 bp), the resulting NRL values (176.7 ± 24.1 bp for PN1 and 188.6 ± 22.6 bp for PN56) are close to those observed by analysis of MNase-digested PN1 and PN56 chromatin (184 ± 3.8 and 193 ± 2.6 bp, respectively, Fig. 1F), while the standard deviations in the distance between individual nucleosomes resolved by cryo-ET are dramatically higher than between the DNA gel measurements of averaged NRLs.

As with distance *D*, the measured DNA linker lengths (*L*) distribution showed a notable increase in the frequency of very short (∼10 bp) and negative length (<0 bp) linkers in PN1 (Fig. 4D and E). The negative length linkers form when two cores closely overlap, such as *L*_7_, *L*_8_, and *L*_11_ in Fig. 4B, comprising 10.7% of all linkers in PN1. The very short linkers are found in the same arrays with the very long ones (e.g. *L*_4_ and *L*_5_), thus generating a very strong linker length heterogeneity in PN1. In PN56, the fraction of negative length linkers is much smaller (3.2% of all linkers), still very short linkers and long linkers co-exist in the same arrays (Supplementary Table S3).

The observed differences in linker length distribution rather than core DNA unpeeling are also prominent in analysis of *C* and *L* average values per each individual array (Fig. 4F and G). Remarkably, the distribution of *L*_av_ in PN56 is biphasic (Fig. 4G), which is consistent with a more diffuse nucleosome ladder for PN56 observed by MNase digestion (Fig. 1E). The two peaks of *L*_av_ distribution at 40 and 50 bp (red arrows in Fig. 4G) are close to integer numbers of DNA double helical turns (4 and 5) that facilitate compact two-start zigzag folding in reconstituted nucleosome arrays [23–25]. In contrast, the major peak in PN1 *L*_av_ distribution, ∼35 bp, is close to non-integer (3.5) number of DNA turns known to weaken chromatin folding [42, 43].

In comparison, the *C*_av_ values do not differ significantly between PN1 and PN56 (Fig. 4F), and there is no correlation between *C* and *L* values within PN1 and PN56 nucleosomes and arrays (Supplementary Fig. S2B–E), indicating that the shorter linkers are not associated with DNA unpeeling. We also do not observe any bias associated with analyzed nucleosome array size (Supplementary Fig. S2F–I).

Thus, during the process of retina maturation and exit from cell cycle major organizational changes in the primary nucleosome chain organization occur. These changes involve not only an increase in the average nucleosome linker length but also a significant nucleosome redistribution and a striking decrease in the fraction of nucleosomes juxtaposed at a very short distance.

### Chromatin from mature retina undergoes a stronger secondary level compaction

**Figure 5.**
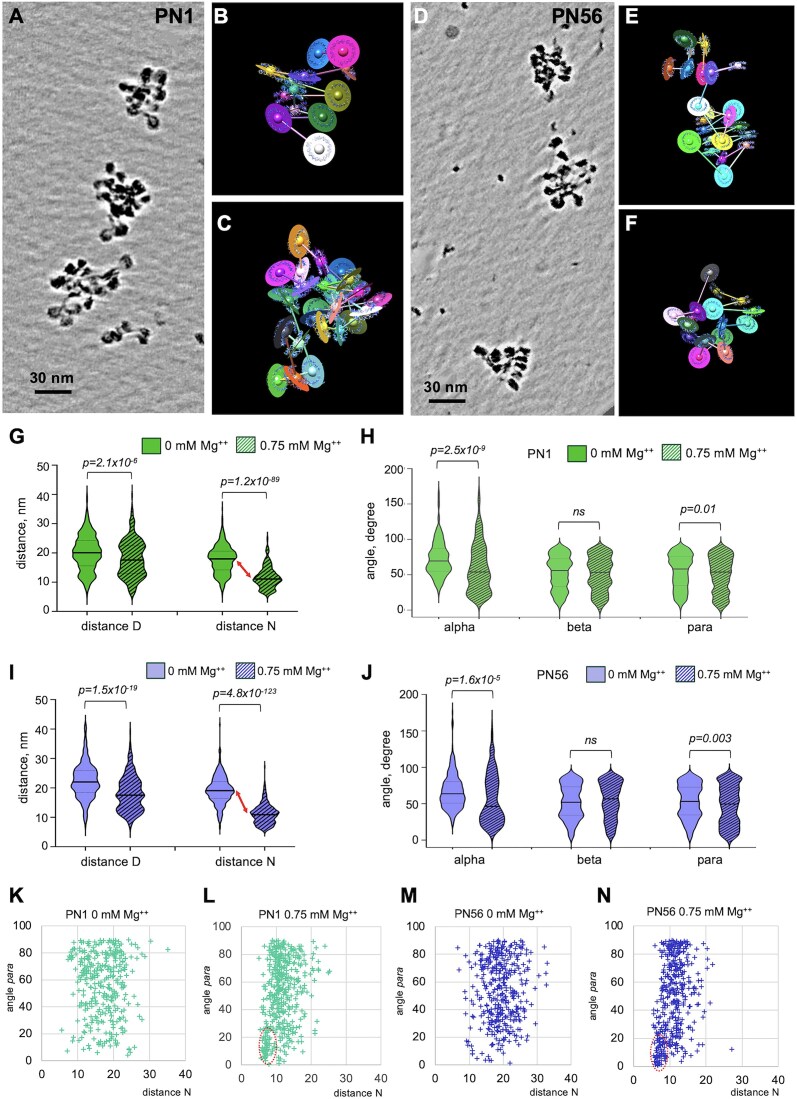
Cryo-ET and stereological analysis of immature and mature retina chromatin folding. (**A**) Cryo-ET tilt series of the PN1 chromatin vitrified at 0.75 mM Mg^2+^(TS_3_2) were processed by deep learning denoising and shown as a composite of Z-series slices in IMOD. Cropped images of the PN1 chromatin shown in panel (A) were processed to generate CAP models CAP_3_11 (**B**) and CAP_3_10 (**C**). (**D**) Cryo-ET tilt series of the PN56 chromatin vitrified at 0.75 mM Mg^2+^(TS_11_2) were processed by deep learning denoising and shown as a composite of Z-series slices in IMOD. Cropped images of the PN56 chromatin shown in panel (D) were processed to generate CAP models CAP_11_15 (**E**) and CAP_11_14 (**F**). Violin plots of distances *D* and *N* (**G**) and angles *α*, *β*, and *para* (**H**) obtained for arrays of PN1 nucleosomes vitrified at 0 mM Mg^2+^ [plain green shapes, *n* = 529 (*D*), 570 (*N*), 468 (*α*), 530 (*β*), and 570 (*para*)] and arrays of nucleosomes vitrified at 0.75 mM Mg^2+^ [crossed green shapes, *n* = 334 (*D*), 431 (*N*), 284 (*α*), 345 (*β*), and 430 (*para*)]. Violin plots of distances *D* and *N* (**I**), and angles *α*, *β*, and *para* (**J**) obtained for arrays of PN56 nucleosomes vitrified at 0 mM Mg^2+^ (plain violet shapes, *n* = 344 (*D*), 380 (*N*), 294 (*α*), 347 (*β*), and 380 (*para*) and arrays of nucleosomes vitrified at 0.75 mM Mg^2+^ [crossed violet shapes, *n* = 310 (*D*), 399 (*N*), 249 (*α*), 312 (*β*), and 399 (*para*)]. Two-dimensional plots showing distributions of the internucleosomal angle *para* versus the distance *N* for PN1 nucleosomes vitrified at 0 mM Mg^2+^ (**K**) and 0.75 mM Mg^2+^ (**L**) and PN56 nucleosomes vitrified at 0 mM Mg^2+^ (**M**) and 0.75 mM Mg^2+^ (**N**).

With reconstituted nucleosome arrays, Mg^2+^ ions at a concentration of 0.5–1 mM (very close to the physiological concentration range of free Mg^2+^ [75]) induce compact chromatin folding into two-start zigzag 30-nm fibers [21, 24]. Since we observed striking changes in linker DNA organization potentially promoting 30-nm fiber formation in mature retina chromatin, we asked whether this chromatin acquires a stronger potential for forming 30-nm fibers. Therefore, we induced condensation of PN1 and PN56 chromatin by adding 0.75 mM Mg^2+^ to achieve maximal compaction without inducing self-association (Fig. 2F and G), conducted cryo-ET (Fig. 5A and D), built CAP models (Fig. 5B, C, E, and F) and examined internucleosome distances *D* and *N*, and the nucleosome plane angles *α*, *β*, and *para* (Fig. 5G–J) as we did before for open chromatin.

Comparative analysis of multiple nucleosomes vitrified at 0 and 0.75 mM Mg^2+^ in PN1 and PN56 chromatin showed that the average distance *D* is reduced modestly, by 10.0% of the original value for PN1 (Fig. 5G) and 9.3% for PN56 (Fig. 5I), consistent with relatively small linker DNA contraction upon condensation and similar to prior observation for human chromatin [62]. In comparison, the average distance *N* is reduced more profoundly, by 34.4% for PN1 and 42.7% for PN56 (Fig. 5G and I), producing a prominent peak at ∼12 nm and a broader peak at ∼6.5 nm that corresponds to the average distance between the perpendicularly proximal nucleosome disks and parallel stacked nucleosomes, respectively [62]. The overall degree of compaction by Mg^2+^ that we assess by the average distance *N* was very close for the human (10.9 nm) and the PN56 mouse chromatin (11.0 nm) with notably less compact PN1 chromatin (11.6 nm).

Distribution of angle *α* shows a sharp and significant increase in average values for PN1 and PN56 chromatin, generating a broad peak at ∼20^o^ (Fig. 5H and J). The angle *α* distributions are similar to that found in compact K562 chromatin but much wider than in compact clone 601 reconstitutes [62]. This observation is consistent with heterogeneous angle *α* populations and likely reflects a variable association with LH H1 that reduces the angles between linker DNA [77, 78]. Angle *β* distributions are very wide in both PN1 and PN56 chromatin and do not change significantly upon chromatin compaction (Fig. 5H and J), indicating that the nucleosome plane orientations remain highly variable during compaction, consistent with the highly heteromorphic chromatin fibers observed by cryo-ET in immature and mature retina chromatin (Fig. 5A–F).

In both types of condensed retina chromatin, the frequency of internucleosomal angle *para* displayed a notable increase at values below 25° consistent with appearance of a fraction of stacked nucleosomes with near-parallel orientation of nucleosome discs (Fig. 5H and J). To reveal associations between the angle *para* and the internucleosomal distance *N*, we plotted their distributions on two-dimensional plots (Fig. 5K–N). These plots show distinct areas below 25^o^ and centered at the distance *N* ∼ 6.5 nm that are prominent at 0.75 mM Mg^2+^ (dashed ovals on Fig. 5L and N) and absent at 0 mM Mg^2+^ (Fig. 5K and M). These areas correspond to tightly stacked nucleosomes [62]. Interestingly, with both PN1 and PN56 retina samples, the number of stacked nucleosomes, 10.7% and 12.5%, respectively, appeared to be about three-fold lower than the number of stacked nucleosomes (34.3%) in human K562 chromatin [62].

Thus, chromatin from mature PN56 mouse retina undergoes a greater degree of secondary level compaction than the immature PN1 retina chromatin, while maintaining high level of structural heterogeneity that is reflective of the intrinsic variability of the nucleosome positions in its primary structure rather than forming regular 30-nm fibers.

### *In situ* crosslinking and characterization of soluble chromatin by cryo-EMANIC

**Figure 6.**
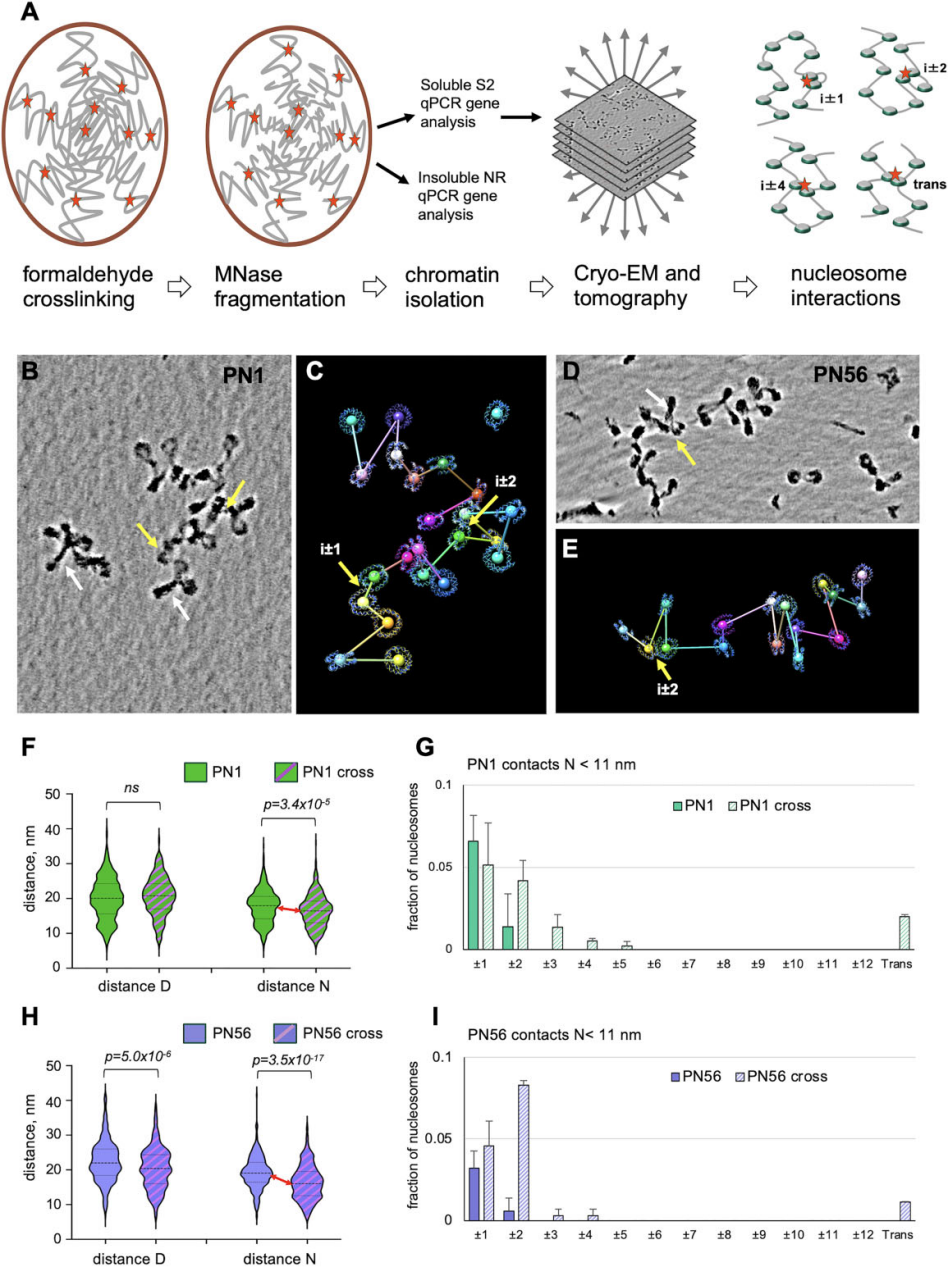
Cryo-EMANIC and analysis of nucleosome contacts *in situ*. (**A**) Scheme of the experimental procedure steps for cryo-EMANIC. *i* ± 1, *i* ± 2, and *i* ± 4 result from intra-fiber internucleosomal crosslinking; trans results from inter-fiber nucleosome crosslinks. (**B**, **D**) Examples of cropped subtomograms of the *in-situ* crosslinked PN1 (B: TSS_8_1) and PN56 (D: TSS_15_2) chromatin vitrified in HNE buffer shown as a composite of Z-series slices in IMOD. The cropped images (B, D) were processed by deep learning denoising (B, D) and used to generate CAP models CAP_7_4 (**C**) and CAP_15_8 (**E**). Planes are not shown to emphasize interactions. Yellow arrows indicate specific juxtaposed nucleosome cores (*N* < 11 nm) resulting from crosslinking. White arrows show nucleosome stems. (**F**) Violin plots of distances *D* and *N* obtained for native PN1 nucleosome arrays [plain shapes, *n* = 529 (*D*), 570 (*N*)] and *in-situ* crosslinked nucleosome arrays [crossed shapes, *n* = 395 (*D*), 446 (*N*)]. (**G**) Distribution of nucleosome contacts at *N* < 11 nm determined for native PN1 nucleosome arrays vitrified at 0 mM Mg^2+^ (plain columns) as a fraction of total nucleosomes (*n* = 527) and *in-situ* crosslinked nucleosome arrays (crossed columns) as a fraction of total nucleosomes (*n* = 397). Error bars represent SD values. (**H**) Violin plots of distances *D* and *N* obtained for native PN56 nucleosome arrays [plain shapes, *n* = 344 (*D*), 380 (*N*)] and *in-situ* crosslinked nucleosome arrays [crossed shapes, *n* = 350 (*D*), 416 (*N*)]. (**I**) Distribution of nucleosome contacts at *N* < 11 nm determined for native PN56 nucleosome arrays vitrified at 0 mM Mg^2+^ (plain columns) as a fraction of total nucleosomes (*n* = 339) and *in-situ* crosslinked nucleosome arrays (crossed columns) as a fraction of total nucleosomes (*n* = 350). Error bars represent SD values.

Controlled crosslinking of proximal nucleosomes by formaldehyde *in situ* preserves elements of chromatin higher-order folding captured before chromatin fragmentation and isolation [28, 30]. To reveal the intrinsic features of chromatin architecture specific to the immature (PN1) and mature (PN56) mouse retina chromatin, we combined our EM-assisted nucleosome interaction capture (EMANIC) [28, 71] with cryo-ET (cryo-EMANIC), which involves crosslinking living cells with formaldehyde, followed by fragmentation of nuclear chromatin by micrococcal nuclease, isolation and unfolding of chromatin fragments, and scoring nearest-neighbor nucleosome interactions by cryo-ET (Fig. 6A). Unlike the previous TEM-based approach, cryo-ET imaging is three-dimensional and free from artifacts caused by attachment to the EM grid and heavy metal staining, which results in prominent “husks” partially coating crosslinked chromatin. This allows the quantification of nucleosome interactions with low background and without any protease treatment to open chromatin as was used before [28].

To monitor the extent of formaldehyde crosslinking, we crosslinked PN1 and PN56 retina with formaldehyde ranging from 0% to 0.4%, isolated the nuclei in TE buffer that causes a dramatic unfolding of the unfixed nuclei, and measured the nuclei areas by fluorescence microscopy. Both PN1 and PN56 nuclei were efficiently crosslinked with 0.3% formaldehyde, causing an almost complete reduction of the nuclear area (Supplementary Fig. S3A–C). With >0.3% formaldehyde, we observed a significant decrease in solubility and therefore used crosslinking with 0.3% formaldehyde that released ∼73% soluble chromatin (Supplementary Fig. S3D and E). We also assessed the gene composition of the soluble and insoluble chromatin fractions by genomic qPCR and observed that while some actively transcribed genes were underrepresented in the solubilized chromatin, genes that represented repressed and condensed chromatin were readily solubilized after 0.3% formaldehyde crosslinking (Supplementary Fig. S3F and G).

Typical cryo-ET denoised images of nucleosome arrays isolated from crosslinked PN1 and PN56 nuclei and corresponding CAP models are shown in Fig. 6B–E. For both PN1 and PN56 chromatin, the *in-situ* formaldehyde crosslinking resulted in a visible increase in the number of closely juxtaposed nucleosomes (yellow arrows in Fig. 6B–E) and nucleosome linker “stems” (white arrows). Stereological measurements showed a significant decrease in the internucleosomal distances *N* induced by Mg^2+^ (Fig. 6F and H). The distance *N* was more prominently reduced for PN56 (by 14.7%) than for PN1 (7.0%) apparently reflecting the more condensed chromatin state in the mature retina cells.

For quantification, we defined direct nucleosome contacts as those occurring at distance *N* < 11 nm (double nucleosome disk radii) between the centroids as we did before [28] and determined relative nucleosome positions in the chain (*i* ± *k*) between the contacting nucleosomes. Figure 6G and I shows the relative abundance of cases where two consecutive nucleosomes are contacting (*i* ± 1), cases involving contacts (loops) over one (*i* ± 2) or more (*i*± >2) nucleosomes separating the crosslinked pair, and cases where two nucleosomes are contacting in-trans, between different arrays. Between the control and formaldehyde-crosslinking samples, the extent of the *i* ± 2 interactions increased most substantially: ∼3-fold for PN1 and ∼14-fold for PN56 (Fig. 6G and I). At the same time, PN56 showed almost all increase due to *i* ± 2 interactions, while PN1 also showed notable increase at *i* ± 3, *i* ± 4, and trans contacts. Contacts at *i* ± 1 did not change substantially for either samples, indicating that while the closely juxtaposed nucleosomes persist in the crosslinked material, no additional folding due to chromatin condensation involves interactions between the nearest neighbor nucleosomes.

Remarkably, the overall contact pattern in PN1 closely resembles that previously observed by EMANIC in interphase HeLa cells, while the one in PN56 is rather similar to reconstituted clone 601 chromatin, showing strong traits of the two-start zigzag [28]. It thus appears that a major structural transition leading to the tight chromatin packing in mature retina originates from a folding according to the two-start zigzag despite the apparent absence of regular and helical 30-nm fibers.

### Mesoscale chromatin modeling predicts that the short and irregular linker length distributions typical of PN1 compromise chromatin compaction

**Figure 7.**
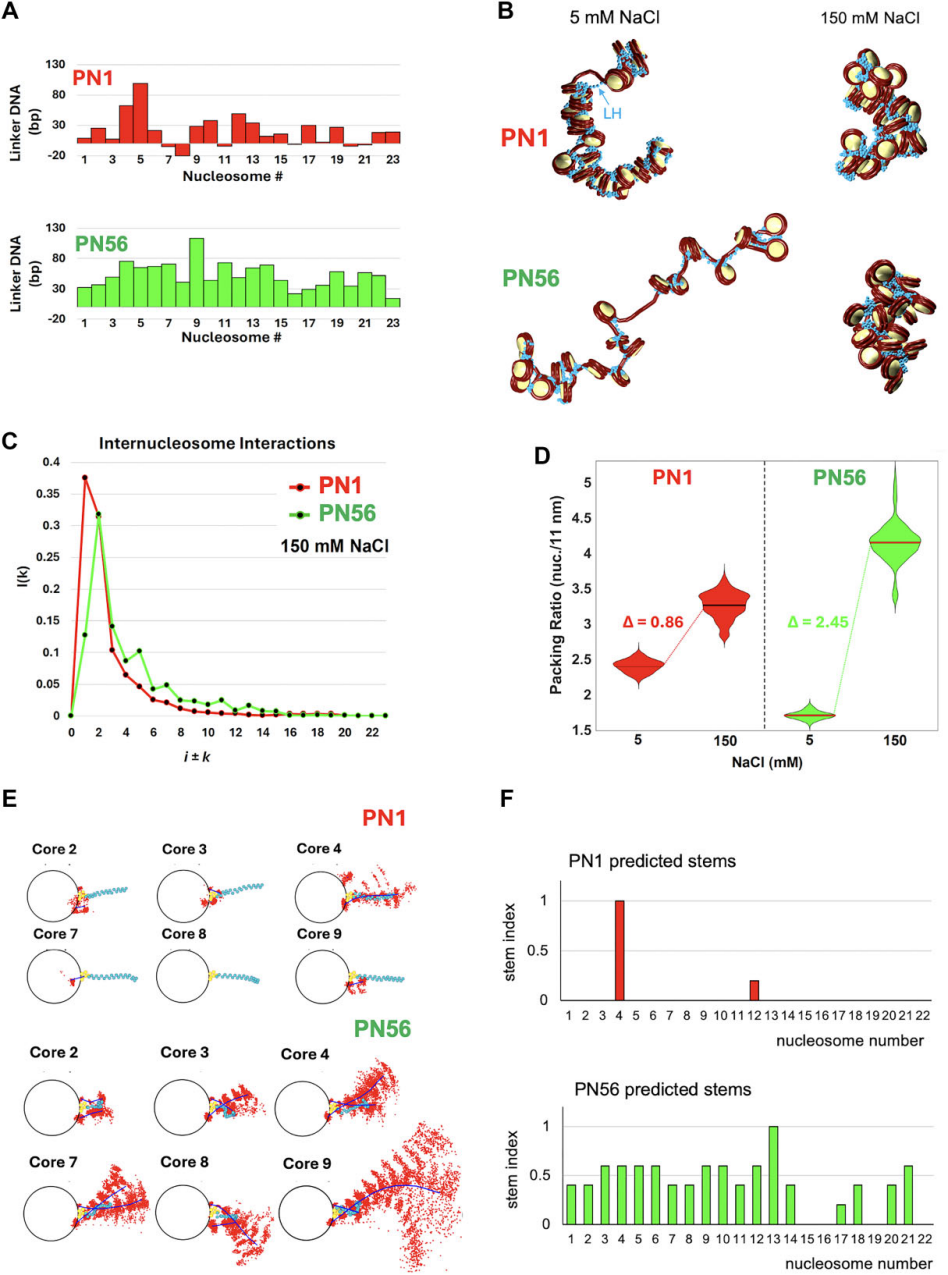
Mesoscale chromatin modeling and structural analysis of native chromatin from immature and mature retina. (**A**) Bar graphs showing linker length *L* measured for a PN1 nucleosome array (red, CAP 1_4) and PN56 array (green, CAP 9_2). (**B**) Representative conformations are shown for PN1 nucleosome arrays (top) and PN56 arrays (bottom) simulated at monovalent salt concentration of 5 and 150 mM NaCl as indicated. (**C**) Nucleosome interaction patterns *i*±*k* are shown for PN1 model (red) and PN56 model (green) with 1 LH per nucleosome at monovalent salt concentration of [NaCl] = 150 mM. (**D**) Chromatin packing ratios measured as the number of nucleosomes per 11 nm of the main fiber axis are shown for PN1 nucleosome arrays (red) and PN56 arrays (green) simulated at monovalent salt concentration of 5 and 150 mM NaCl as indicated. (**E**) Fan plots representing, the linker DNA cumulative and average positional distribution across a single trajectory along each nucleosomal plane for selected PN1 and PN56 nucleosomes with shorter linkers (in PN1) and longer linkers (in PN56) modeled with 1 LH per nucleosome at monovalent salt concentration of [NaCl] = 150 mM. (**F**) Bar graphs showing distributions of the predicted nucleosome stem formation index for the PN1 and PN56 nucleosome arrays modeled with 1 LH per nucleosome at monovalent salt concentration of [NaCl] = 150 mM.

Mesoscale chromatin modeling at nucleosome resolution can assess multiple nucleosome chain configurations to detect histone tail-mediated interactions and other conformational details of internucleosomal interaction patterns and chromatin compaction [69, 70]. For modeling, we selected two long arrays (>20 nucleosomes) that showed the largest differences between PN1 and PN56 (labeled by red arrows at Supplementary Fig. S2F–I). The PN1 array contained 23 nucleosomes, and from the longer PN56 array, we took the first 23 nucleosomes to equalize the array sizes. We developed two chromatin models for the two selected PN1 and PN56 arrays (Fig. 7A) to investigate the relationship between linker length distributions and chromatin condensation. For chromatin folding experiments, we modeled chromatin fibers with fully saturated LH (1 LH per nucleosome consistent with LH stoichiometry in mouse retina [48]) and simulated chromatin folding at 5 and 150 mM NaCl, as well as in the presence or absence of 1 mM MgCl_2_ (Fig. 7B and Supplementary Fig. S4).

As shown on Fig. 7B–D, at physiological salt, the two types of fibers predicted for PN1 and PN56 chromatin strikingly recapitulate the experiments: predominantly *i* ± 1 interactions in PN1 versus *i* ± 2 contacts in more compact PN56 (Fig. 7B and C, and Supplementary Fig. S4). Remarkably, at low salt, the PN56 array appears to be much less compact than PN1, mostly due to disordered long linkers. At physiological salt, the long linkers fold compactly in the PN56 fiber, exhibiting a much stronger total amplitude for the salt-dependent chromatin packing measured as the number of nucleosomes per 11 nm unit length (Fig. 7B and D). Our modeling is thus consistent with experimental observation of the much higher amplitudes of PN56 nuclear chromatin compaction (Fig. 1C) and chromatin compaction by *in situ* crosslinking (Fig. 6F and H) and helps detect the underlying mechanism.

Namely, by plotting the configurations of the nucleosome linkers exiting and entering the nucleosomes with different linker lengths (Fig. 7E and F, and Supplementary Fig. S5), we see that the longer nucleosome linkers tend to form in a characteristic stem where the distance between a pair of beads located at the same position on the two linker DNAs is <2.5 nm. Stem formation depends critically on linker DNA length. For nucleosomes with both linkers >26 bp, linker DNA stems form narrowing, the positional distribution of both linkers and promoting the *i* ± 2 contacts between the neighboring nucleosomes. In contrast, shorter linkers cannot form stems (Fig. 7F) and therefore reduce *i* ± 2 interactions and decrease overall fiber compaction for PN1 at physiological salt (Fig. 7B). From tail interaction analysis of PN1 versus PN56 systems at two salt conditions in Supplementary Fig. S6, we also suggest that PN1 with short linkers has less tail–DNA interactions (green and orange) but more tail–tail (purple) and tail–nonparental core (blue) interactions than the PN56 system of longer linkers. Thus, the high frequency of short linkers critically inhibits nucleosome chain folding and may explain the unfolded chromatin states observed in immature retina and possibly other proliferating cells. These features are in dramatic contrast with more ordered zigzag in mature chromatin.

## Discussion

**Figure 8.**
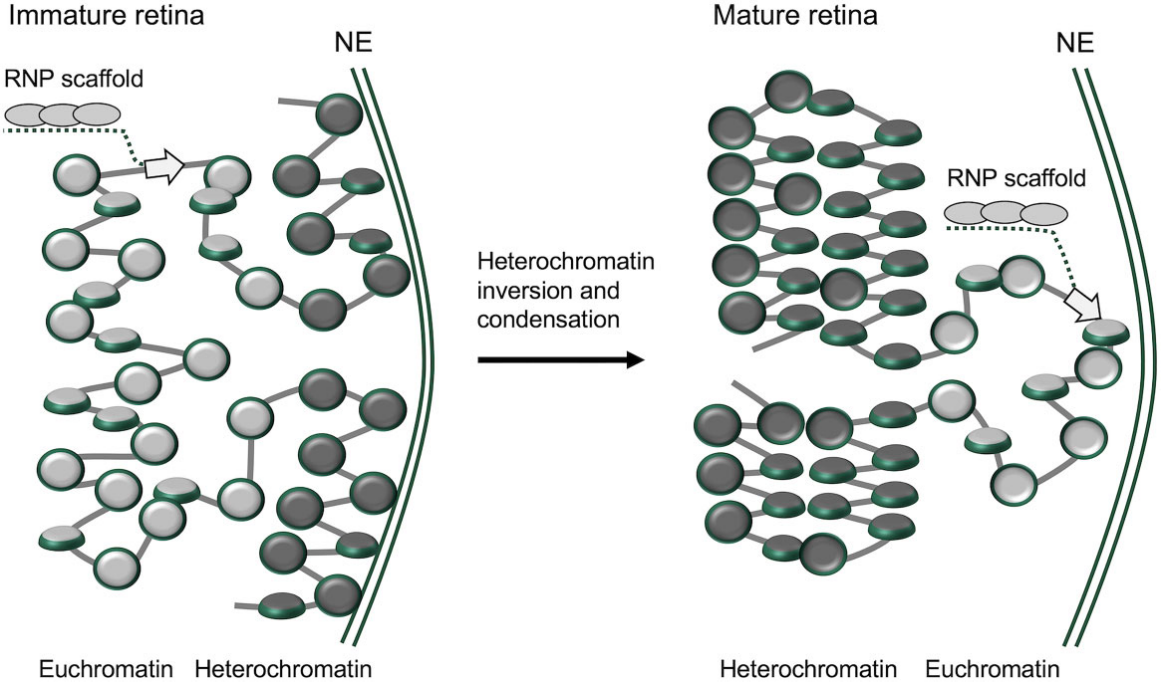
Schematic model of chromatin higher-order folding controlled by nucleosome spacing in naturing mouse retina. Schematic drawing of nucleosome chain folding in immature (left) and mature (right) mouse retina cells chromatin implies that the closely spaced di- and tri-nucleosomes render nucleosome arrays more open in immature rod photoreceptors. During retina maturation, heterochromatin fraction increases, number of closely-spaced nucleosomes decreases, and the chromatin fibers become globally condensed via tight accordion-like zigzag folding. This mechanism may be a general way of maintaining a more open chromatin to ensure high epigenetic plasticity in immature and undifferentiated cells and specialized gene expression programs in differentiated and mature cells. NE: nuclear envelope.

One of the most intriguing natural biological phenomena associated with cell differentiation and tissue development involves accumulation of abundant facultative heterochromatin and its spatial segregation from euchromatin [79, 80], which is strongly manifested in mature mammalian retina. Mammalian retina is a part of the central nervous system that has been studied extensively in relation to general epigenetic and chromatin-mediated mechanisms of neuronal differentiation [48, 49, 54]. Initially, based on the previous observation of 30-nm structural signal in mature retina [54] and experiments with reconstituted chromatin with various NRLs [20, 71, 81], we expected that the increased NRL and levels of histone H1 in mature retina [48] would lead to formation of condensed 30-nm chromatin fibers, while the shorter nucleosome repeat would promote an open nucleosome zigzag. However, our cryo-ET analysis revealed remarkable structural heterogeneity incompatible with any helical structures in condensed chromatin of either immature or mature retina (Fig. 2). With open nucleosome arrays, the linker DNA length is strikingly irregular in the immature retina. We propose that in the nucleus of immature retina cells, the open chromatin is maintained by the very frequent short linkers that effectively inhibit compact nucleosome folding (Fig. 8, left). Additionally, external nuclear structures such as ribonucleoprotein scaffolds [82] may support the open chromatin state while the nuclear lamina [55] tethers heterochromatin at the nuclear periphery in immature retina. During retina maturation, nucleosomes reorganize to acquire longer linker DNA lengths (Fig. 4D and G) that promote formation of discontinuous two-start zigzag structures and their local accordion-like compaction without forming extended 30-nm fibers (Fig. 8, right). At this stage, the attachments between heterochromatin and the nuclear lamina are disrupted, allowing heterochromatin to congregate near the center of the nucleus [55]. It thus appears that during retina cell maturation and chromatin inversion there is no abrupt transition from irregular chromatin to regular 30-nm fibers. Rather, the increasing linker DNA length and decreasing fraction of overlapping nucleosomes allow the chromatin to gradually increase the two-start zigzag features and fold more compactly, despite residual short linkers that interrupt the continuity of the chromatin fiber.

Remarkably, a significant fraction of nucleosomes in immature retina chromatin has negative linker length values (e.g. L7, L8, and L11 in Fig. 4B) and thus contains partially overlapping two or three nucleosome cores. Such overlapping nucleosomes were initially observed as a result of chromatin remodeling by SWI/SNF *in vitro* [83]. With native chromatin, overlapping nucleosomes were observed by cryo-ET in human HeLa chromosomes [61] and *Drosophila* embryos [84]. Another interesting example of an array of partially overlapping nucleosomes has been reconstituted from telomeric DNA repeats and shown to fold into a non-conventional columnar structure [44]. By genome-wide mapping, overlapping nucleosomes were found to be enriched at transcriptional start sites in human HeLa cells [85, 86] and actively transcribed genes in murine embryonic stem cells [87]. Structures of overlapping di- and tri-nucleosomes reconstituted from nucleosome positioning DNA and mixture of histone octamers and hexamers lacking one histone H2A/H2B dimer have been solved by X-ray crystallography [85] and cryo-EM [86].

Currently, our cryo-ET resolution does not allow to determine whether the short-linker and overlapping nucleosome composition in mouse retina chromatin deviates from the canonical histone octamer. However, the fact that the nucleosomes from immature retina do not show an increased DNA unpeeling (Fig. 4C and F, and Supplementary Fig. S2B–E) argues against a significant structural deformation of the short-linker nucleosomes. In the future, developing a biochemical fractionation of the short-linker and long-linker nucleosome arrays may help to reveal changes in core histone stoichiometry or identify specific histone variants and modifications contributing to regulation of chromatin folding. Another limitation of this study is that we analyze total chromatin isolated at two developmental stages without specific selection of euchromatin and heterochromatin fractions. In the future, application of cryo-ET technique to resolve retina chromatin organization *in situ* may further clarify the association of partially overlapping and short-linker nucleosomes with euchromatin and heterochromatin compartments.

Cryo-ET analysis of mouse retina chromatin reveals several new features of the partially overlapping nucleosomes. First, they are abundant in immature retina, and their frequency (10.7% of total linkers in PN1) appears to be much higher than that expected from transcription start sites. Second, the extent of the nucleosome overlap is variable (Fig. 4E), and the strictly overlapping nucleosomes (with negative-length linkers) do not make a structurally homogeneous unit but rather include a group of two to three closely juxtaposed nucleosomes with very short linkers. Third, the overlapping nucleosomes can also be found in the mature retina, where they are less abundant and often interspersed, within the same array, with the longer-linker nucleosomes containing stems.

The unprecedented heterogeneity of linker DNA length makes the native chromatin folding incompatible with previous chromatin fiber models based on regular helical nucleosome arrangements and average NRL [46]. Here, to account for the native nucleosome positional heterogeneity, we generated for the first time nucleosome-resolution chromatin models with individual nucleosome linker length variations derived from native mouse retina chromatin. Our simulated chromatin fiber systems of PN1 and PN56, remarkably consistent with experiments, suggest a new mechanism: partially overlapped nucleosomes are sufficient to perturb chromatin two-start zigzag folding in immature mouse retina. Interestingly, not just the overlapped nucleosomes, but all nucleosomes with rather short linkers, below 26 bp, limit formation of the nucleosome stem and thus impede histone H1-induced chromatin folding (Fig. 7). That the length of the linker DNA perturbing stem formation (<26 bp) is very close to those constrained by histone H1 positioned near the dyad axis of a chromatosome [45, 88] and directly contacting histone H1 C-tail domain [89] is consistent with perturbance of stem formation being the main mechanism by which the short linkers inhibit chromatin folding.

What are the factors that regulate nucleosome spacing and cause partial nucleosome overlapping in immature retina? And what leads to the increased nucleosome spacing upon retina cell maturation? In living cells, nucleosome spacing is dynamically regulated by ATP-dependent chromatin remodelers [90]. As we discussed earlier, the overlapped nucleosomes result from the activity of nucleosome remodelers SWI/SNF *in vitro* and are attenuated by murine SWI/SNF homolog BRG1 (Smarca4) and ISWI homolog SNF2H (Smarca5) in mouse embryonic stem cells [87]. Remarkably, Smarca4 is important for cell integrity in early retina progenitors [91], and SmarcA5 is essential for retinal cell proliferation and photoreceptor maintenance [92]. Another factor essential for maintenance of the NRL is histone H1 [93], whose expression is increased in mature mouse retina, and its partial knockout promotes antigenic exposure but not unfolding of the facultative heterochromatin [48]. Whether the chromatin remodelers and/or histone H1 levels control the number of overlapped nucleosomes in developing retina remains to be determined.

Together, the observation of abundant overlapping nucleosomes due to short and irregular nucleosome linkers in immature retina, with development-mimicking chromatin models predicting compromised chromatin folding, define a new mechanism for developmentally regulated control of chromatin structure in mouse retina. The precise nature of nucleosome positioning (or position-randomizing) factors and how such mechanisms affect chromatin compaction in other cells and tissues are exiting areas for future research. Indeed, for mouse ES cells, HeLa, and *Drosophila* embryos where the partially overlapped nucleosomes have been associated with open chromatin, new mechanistic questions on the role of nucleosome distribution in the chromatin folding plasticity and epigenetic regulation naturally emerge.

## Supplementary Material

gkaf457_Supplemental_Files

## Data Availability

The raw cryo-EM tilt series files (*.mrc), the data folders containing Chimera stereological model files (**.py) paired with cropped subtomogram files (**.mrc), and the resource needed to visualize chromatin configurations simulated by mesoscale modeling are available on the Dryad data depository at the following link: https://doi.org/10.5061/dryad.bcc2fqzpz. Resource for synthesis of cryo-electron data from PDBs (cryo-TomoSim) is publicly available at Zenodo depository at the following link: https://doi.org/10.5281/zenodo.8234233. Any additional information required to reanalyze the data reported in this paper is available from the corresponding author upon request.
