# Supplementary material for: Compromised two-start zigzag chromatin folding in immature mouse retina cells driven by irregularly spaced nucleosomes with short DNA linkers
Source: Nucleic Acids Res. 2025 Jun 4;53(10):gkaf457. doi: 10.1093/nar/gkaf457 (PMC12135184; doi:10.1093/nar/gkaf457)
Supplement: gkaf457_Supplemental_Files [file gkaf457_supplemental_files.zip › Supplemental_Text_Figures.pdf]

Supplemental Material

Compromised 2-start zigzag chromatin folding in immature mouse retina cells driven by irregularly spaced nucleosomes with short DNA linkers.

Brianna Kable[1], Stephanie Portillo-Ledesma[3], Evgenya Y. Popova[2], Nathan Jentink[1], Matthew Swulius[1], Zilong Li[3], Tamar Schlick[3], and Sergei A. Grigoryev[1]*

[1,2] Penn State University College of Medicine, Dept. Biochemistry & Molecular Biology[1], and Dept. of Neural and Behavioral Sciences[2], 500 University Drive, Hershey, PA 17033; [3]Department of Chemistry and Simons Center for Computational Physical Chemistry, New York University, New York, NY 10003; [4]Courant Institute of Mathematical Sciences, New York University, 251 Mercer Street New York, New York 10012 and New York University-East China Normal University Center for Computational Chemistry, New York University Shanghai, Shanghai 200122, China.

Content:

1. Supplemental Mesoscale Modeling Methods text
2. Supplemental Figures 1 – 6.

Supplemental Mesoscale Modeling

Our chromatin mesoscale model combines coarse-grained representations of nucleosome cores, histone tails, linker DNA, and LHs within chromatin fiber arrays. Each chromatin element is coarse grained at a different level of resolution. The nucleosome cores are treated as disks with 300 Debye-Hückel discrete surface charges calculated by the DiSCO algorithm to mimic the electrostatic environment of the atomistic nucleosome (1). The N-terminal tails of histones H2A, H2B, H3, and H4, and the C-terminal tail of H2A are coarse grained as 5 residues per bead and attached to the nucleosome surface; each bead has a charge equal to the sum of the charges of the 5 residues that compose the bead (2). In total, there are 50 tail beads per core: 16 for H3, 10 for H4, 14 for H2A, and 10 for H2B. Similarly, LH is coarse grained as 28 beads with a resolution of 5 residues per bead, where 6 fixed beads describe the globular head (GH) and 22 flexible beads describe the long C-terminal domain (CTD) (3). The adjustment of charges on each LH bead for different concentrations of monovalent salt is calculated by the DiSCO algorithm. The linker DNA connecting nucleosomes is treated with a combined worm-like chain and bead model (4). The length of the segments connecting beads is $l_0 = 3$ nm, which corresponds to a resolution of 8.8 bp based on $N_{bp} = l_0/rise$, where rise is 0.34 nm, the distance between consecutive bps in a B-DNA. Each linker DNA bead has a salt-dependent charge calculated based on the Stigter procedure (5).

The energy function of the model includes stretching, bending, and twisting local terms for the linker DNA, stretching and bending terms for the tails and LHs, and electrostatic and excluded volume terms among each pair of beads as follows:

$$E(r) = E_S + E_B + E_T + E_{tS} + E_{tB} + E_{lhS} + E_{lhB} + E_C + E_V \, , \tag{1}$$

where $\boldsymbol{r}$ is the collective position vector. Details on each energy term can be found in the supporting information of Li et al. (6).

To obtain configurational ensembles in thermal equilibrium, we perform MC simulations of 20 copies of each PN1 and PN56 fibers started from different random seed and different residual twist DNA value of −12°, 0°, or +12°. Each copy is simulated for 40 million MC steps and the last 10 million steps of each trajectory are used for analysis.

For each system, we calculate the packing ratio as the number of nucleosomes in 11 nm of fiber, based on:

$$P_R = \frac{N_C * 11}{F_L}, \tag{2}$$

where N_C is the number of nucleosomes and F_L is the fiber length calculated by defining an axis that passes through the nucleosome cores. The fiber axis is defined as a three-dimensional parametric curve composed of three piece-wise polynomials created by performing a cubic smoothing spline interpolation (with smoothing parameter of 0.35) to the nucleosomes x, y, and z coordinates. The polynomials are evaluated at each nucleosome to obtain their center position in each direction. Then, the Euclidean distance between every nucleosome i and $i + 2$ across the fiber is calculated as the difference between the vectors defined by the center position in the x, y, and z directions. All Euclidean distances are then summed to obtain the total fiber length.

We also calculate the internucleosome interactions as follows. Two nucleosomes i and j are considered to be in contact if the distance between any element (core or histone tails) of nucleosome i and any element of nucleosome j is less than 2 nm. Nucleosome contact frequencies are calculated for the combined configurational ensemble that contains 2000 configurations, or 100 configurations from each of the 20 individual trajectories. The frequencies are then normalized by the total number of configurations. Contact matrices $I'(i, j)$ are further decomposed into one-dimensional plots $I(k)$ that depict the magnitude of i, $i \pm k$ interactions as follows:

$$I(k) = \frac{\sum_{i=1}^{N_C} I'(i, i \pm k)}{\sum_{j=1}^{N_C} I(j)} \quad (3)$$

where NC is the total number of cores.

For the PN1 and PN56 fibers, both simulated at 5 and 150 mM NaCl, we create fan plots to represent, along each nucleosomal plane, the linker DNA cumulative and average positional distribution across a single trajectory. We determine the position vector of each DNA bead, $\mathbf{d}_{ij}$, in the frame of reference of the parental nucleosome core with center of mass position r_i and orientation $\{\mathbf{a}_i, \mathbf{b}_i, \mathbf{c}_i\}$. The projected distribution in 3D is then denoted by $\mathbf{d}_{ij} = \{\mathbf{d}'_{ij,x}, \mathbf{d}'_{ij,y}, \mathrm{d}'_{ij,z}\}$, where $\mathbf{d}'_{ij,x} = \mathbf{a}_i \cdot (\mathbf{d}_{ij} - \mathbf{r}_{ij})$, $\mathbf{d}'_{ij,y} = \mathbf{b}_i \cdot (\mathbf{d}_{ij} - \mathbf{r}_{ij})$, and $\mathbf{d}'_{ij,z} = \mathbf{c}_i \cdot (\mathbf{d}_{ij} - \mathbf{r}_{ij})$, and the projected distribution along the nucleosome plane is given by $\mathbf{d}_{ij} = \{\mathbf{d}'_{ij,x}, \mathrm{d}'_{ij,y}\}$.

To assess quantitatively the degree of stem formation in the cores of PN1 and PN56, we measure the distances between the average positions of each pair of beads on the two linker DNAs associated with each core. That is, if any distance between a pair of corresponding beads located at the same position on the two linker DNAs i and j, i.e., i1 and j1, i2 and j2, and so on, is less than 2.5 nm, we consider that these beads contribute to a stem formation. Based on the total number of bead pairs contributing to the stem formation, we calculate a stem index that ranges from 0 to 1 through normalization. We perform this calculation using the average position of the DNA beads over a single trajectory of 40 million Monte Carlo steps, the same trajectory used for calculating the linker fan plots of Figure 7E and Suppl. Figure S5.

For the PN1 and PN56 ensembles of 2000 chromatin configurations at 5 and 150 mM NaCl, we calculate the fraction of configurations in which each tail *t* (*t*=H2A–N-terminal, H2A–C-terminal, H2B–N-terminal, H3–N-terminal, and H4–N-terminal) is 'in contact' with another chromatin element *e* (*e*=core, DNA, or tails of a separate nucleosome).

We construct a two-dimensional matrix, where each matrix element T' is defined as:

$$T'_{(t,e)} = mean\left[\frac{1}{N_C N_e}\sum_{i \epsilon I_C}\sum_{j_e=1}^{N_e}\delta_{i,j_e}^{t,e}(\mathrm{M})\right] \quad (4)$$

with $\delta_{i,j_e}^{t,e}(\mathrm{M}) = \begin{cases} 1 \; if \; contact \\ 0 \; otherwise \end{cases}$

where NC is the total number of nucleosomes, Ne is the total number of chromatin elements, IC is a specific nucleosome along the chromatin fiber, and M is a specific chromatin configuration.

We then normalize the interactions as:

$$T_{(t,e)} \stackrel{\text{def}}{=} \frac{T'_{(t,e)}}{\sum_{e'=1}^{N_e} T'_{(t,e')}} \quad (5)$$

In each specific configuration M, we consider a t-kind tail of nucleosome *i* to be either free or in contact with only one Ne chromatin element based on the shortest distance between their beads and a cutoff of 2 nm.

References

1. Zhang, Q., Beard, D.A. and Schlick, T. (2003) Constructing irregular surfaces to enclose macromolecular complexes for mesoscale modeling using the discrete surface charge optimization (DISCO) algorithm. *J Comput Chem*, **24**, 2063-2074.
2. Arya, G., Zhang, Q. and Schlick, T. (2006) Flexible histone tails in a new mesoscopic oligonucleosome model. *Biophys J*, **91**, 133-150.
3. Luque, A., Collepardo-Guevara, R., Grigoryev, S. and Schlick, T. (2014) Dynamic condensation of linker histone C-terminal domain regulates chromatin structure. *Nucleic acids research*, **42**, 7553-7560.
4. Jian, H., Vologodskii, A.V. and Schlick, T. (1997) A combined wormlike-chain and bead model for dynamic simulations of long linear DNA. *J. Comput. Phys.*, **136**, 168-179.
5. Stigter, D. (1977) Interactions of highly charged colloidal cylinders with applications to double-stranded. *Biopolymers*, **16**, 1435-1448.
6. Li, Z., Portillo-Ledesma, S. and Schlick, T. (2023) Brownian dynamics simulations of mesoscale chromatin fibers. *Biophys J*, **122**, 2884-2897.

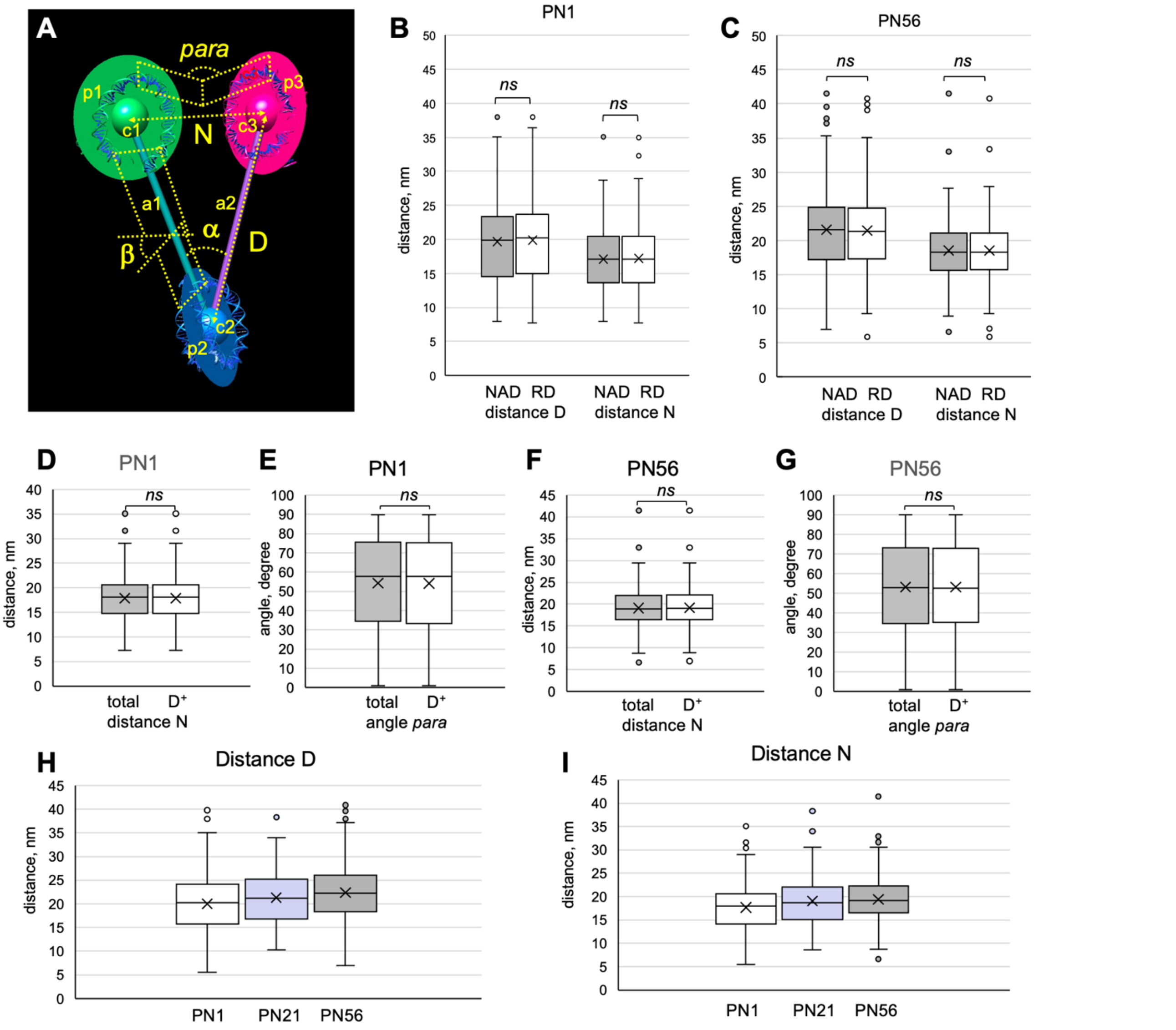

Suppl. Figure S1 (related to Fig. 3). Comparative stereological analysis of nucleosome chain folding in the PN1 and PN56 chromatin.

A: CAP model containing 3 nucleosomes with 3 centroids, 2 axes, and 3 planes shows measurable variables: distance D between consecutive centroids c2 and c3; distance N between nearest centroids (c1 and c3); angle α between consecutive axes (a1 and a2); angle β between consecutive planes (p1 and p2); angle *para* between nearest planes (p1 and p3).

B, C: Box and whiskers plots of distances D and N obtained for arrays of PN1 (B) and PN56 (C) nucleosomes vitrified in HNE buffer and processed either by nonlinear anisotropic diffusion in IMOD nad_eed_3d -n 30 -f -k (NAD, left graphs) or by Regression denoising in Dragonfly (RD, right graphs) show a full agreement between the two independent methods of denoising.

D, E: Box and whiskers plots of distances N (D), and angles *para* (E) obtained for total PN1 nucleosomes vitrified in HNE (total, n = 560) and the nucleosomes with confirmed distances D (D^+, n =530).

F, G: Box and whiskers plots of distances N (F), and angles *para* (G) obtained for total PN56 nucleosomes vitrified in HNE (total, n = 372) and only the nucleosomes with confirmed distances D (D^+, n =328).

H, I: Box and whiskers plots of distances D (H) and N (I) obtained for arrays of postnatal day 1 (PN1), postnatal day 21 (PN21) and postnatal day 56 (PN56) mouse retina chromatin vitrified in HNE buffer show a gradual increase in the internucleosomal distances during mouse retina development.

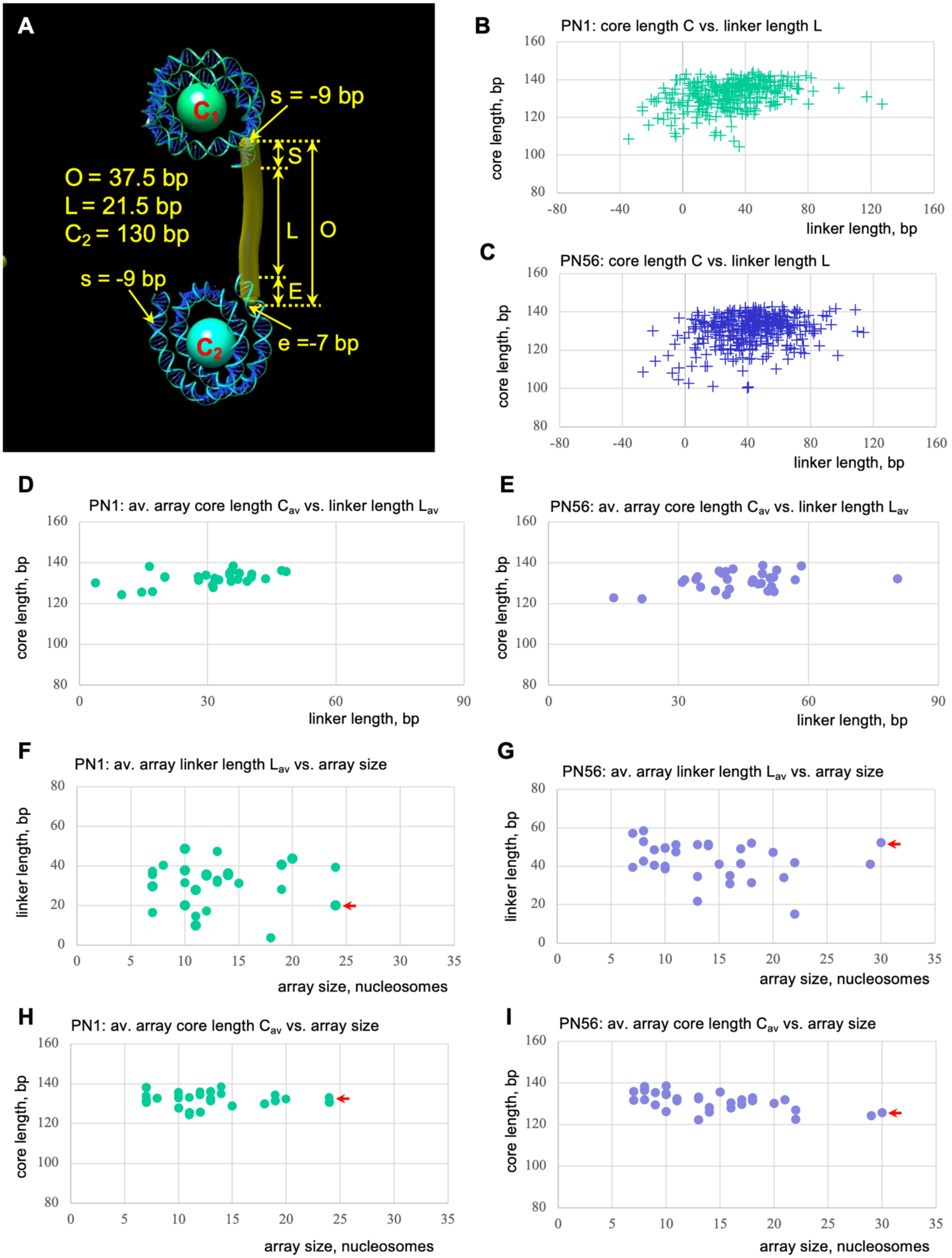

Suppl. Figure S2 (related to Fig. 4). Nucleosome core (C) and linker (L) DNA length distribution in individual nucleosomes and arrays.

A: Linker tracing (LT) model containing two nucleosomes with two centroids, connected by one linker manually traced with Chimera Volume Tracer tool. Total multi-segment open DNA linker lengths (O) are

measured by Chimera "measure pathlength sel" tool. The open DNA regions are exiting and entering the fitted nucleosomes at certain positions within the 146 bp nucleosome core: start (s) and end (e) indicated by arrows. The unpeeled nucleosome core DNA segments (S and E) flanking the traced linker are subtracted from the total open DNA length (O) to give the linker DNA length (L) value. The unpeeled nucleosome core DNA segments (between the s and e points and the end of the 146 bp core DNA) are subtracted from the nucleosome core DNA length (146 bp) resulting in the constrained core DNA length (C).

B, C: Two-dimensional plots showing distributions of the core DNA lengths C vs. linker DNA lengths L for individual nucleosomes. PN1: green crosses, n = 300; PN56: violet crosses, n = 371.

D, E: Two-dimensional plots showing distributions of the average core DNA lengths C_{av} vs. average linker DNA lengths L_{av} for each PN1 and PN56 nucleosome arrays.

F, G: Two-dimensional plots showing distributions of the average linker DNA lengths L_{av} vs. array size (in nucleosomes) for each PN1 and PN56 nucleosome arrays.

H, I: Two-dimensional plots showing distributions of the average core DNA lengths C_{av} per array vs. array size (in nucleosomes) for each PN1 and PN56 nucleosome arrays.

Red arrows on panels F-I indicate the arrays chosen for mesoscale modeling. PN1: dots, n = 27; PN56: violet dots, n = 31.

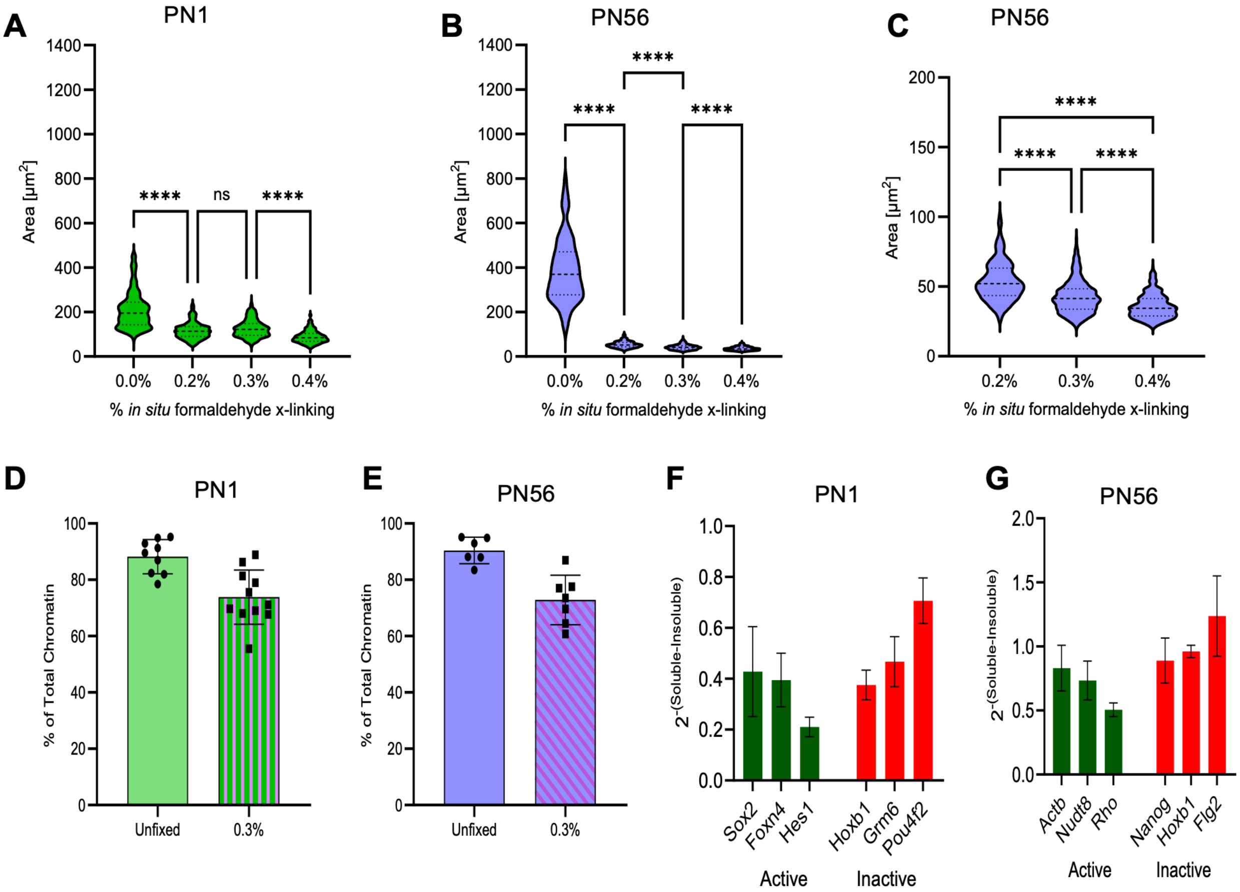

H

Gene	Forward Primer	Reverse Primer
**Hes1**	CAT CCT TTT ACT TCT GTC TAC TGC C	GTC ATG AAG CCT TCA TCG G
**Sox2**	AAA GCA CTC TAG TAA AAG CAA GTC C	GGT GTA CTA TTA CGC TCA ACA CG
**Foxn4**	GAA CTT TGA TCC TGC TGC AAG	CAA GGG TTC TAG GAA ATG GTC C
**Pou4f2**	CAT GTG CTG CCG AAT GC	CGC CAG TCT GAG TGT GTG
**Grm6**	CTG CTG CTT GCT CTC TCC	CTAC ACC GTG AAG ATG TTC AAC
**Hoxb1**	CAG AGG CTG GCT TAC GAG AC	TGG TTG AGG CTT GCT TGA GG
**Actb**	CCA TTC AAT GTC TCG GTT ACT AGG	ACT CTC TGT CCA TTC AAT CCA GG
**Rho**	AAC ACA GTG CAA GAC ATG ACC	TGC AGA CTG AGG CTG CTC
**Nudt8**	TGA TCC CGA CGA CCA AGA TG	GTG ATA GGA TGA GGG TGG CTC
**Flg2**	TCA GGT CAG TCT TGC TGT GG	CCC ATG CTG CCC ATA GTG AC
**Nanog**	GTA AAG CCT CTT TTT GGG G	CAC CAG CCC TGT GAA TT

Suppl. Figure S3 (related to Fig. 6). In-situ chromatin crosslinking and solubilization for Cryo-ET.
A, B, C: Violin plots show nuclear area distributions of the crosslinked and unfolded PN1 and PN56 nuclei crosslinked *in situ* by the indicated amounts of formaldehyde and placed in a low-salt TE buffer to induce nuclear chromatin unfolding, Note the inhibition of unfolding by the gradually increasing amount of formaldehyde.
D, E: Bar graphs showing % of total chromatin (by DNA spectrophotometry) released in soluble form after MNase digestion of intact retina nuclei and those crosslinked *in situ* with 0.3% of formaldehyde.
F, G: Genomic quantitative real-time PCR (qPCR) was conducted for selected transcriptionally active and repressed genes from soluble and insoluble chromatin released after MNase digestion of retina nuclei crosslinked with 0.3% of formaldehyde. Bar graphs show ratio of gene enrichment in the soluble vs. insoluble forms.
H: Sequences of oligonucleotide primers for the qPCR.

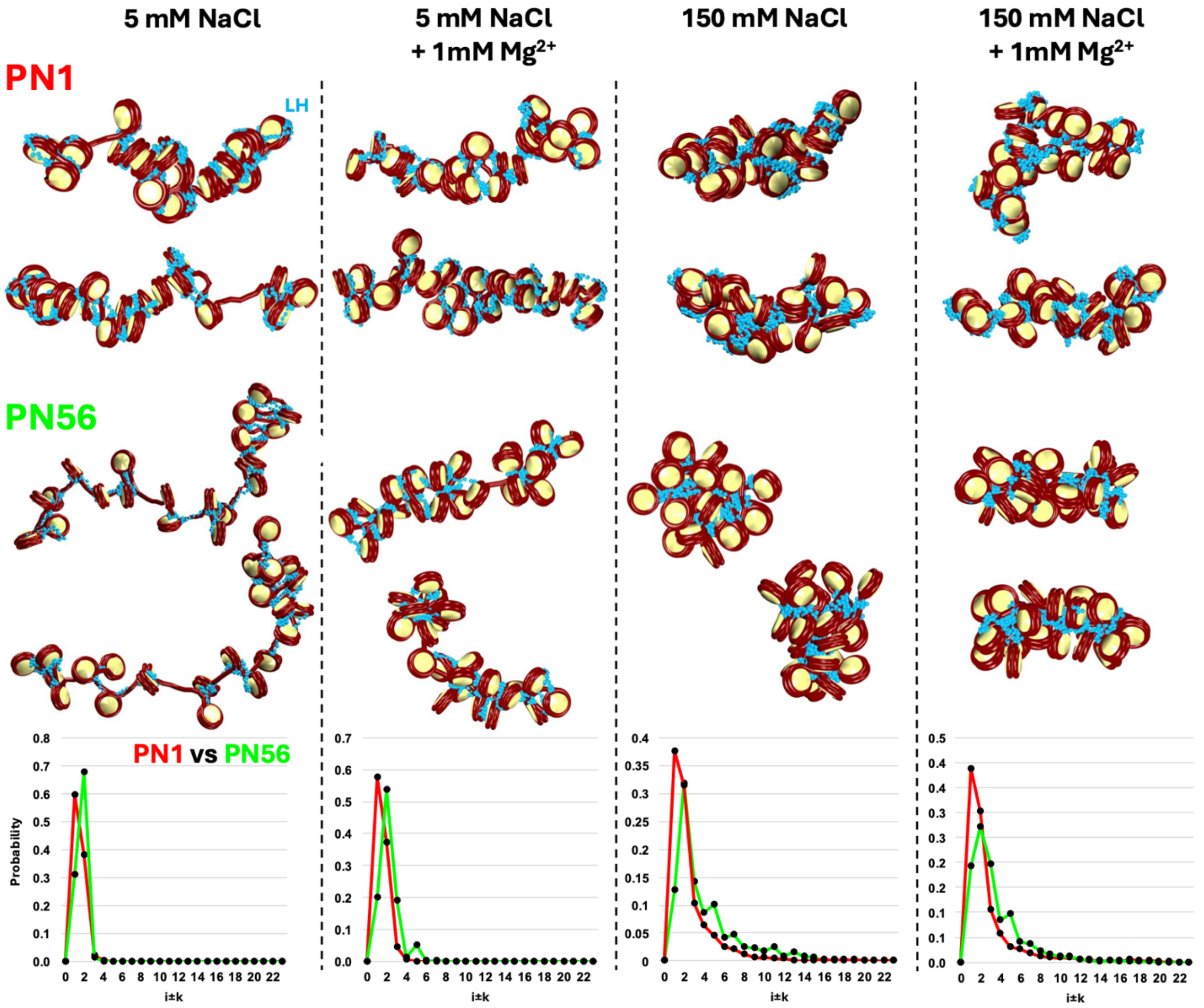

Suppl. Figure S4 (related to Fig. 7). Effect of Mg^{2+} ion on native chromatin folding and nucleosome interactions.

For the PN1 and PN56 systems we show two fiber configurations for each salt condition: 5 mM NaCl; 5 mM NaCl + 1mM Mg^{2+}; 150 mM NaCl; and 150 mM NaCl + 1mM Mg^{2+}. Nucleosomes are shown in light yellow, DNA in dark red, and linker histone (LH) in cyan. At bottom we show at each salt condition the internucleosome interaction patterns $i \pm k$ for PN1 (red) versus PN56 (green) calculated from the 2000-configuration ensemble.

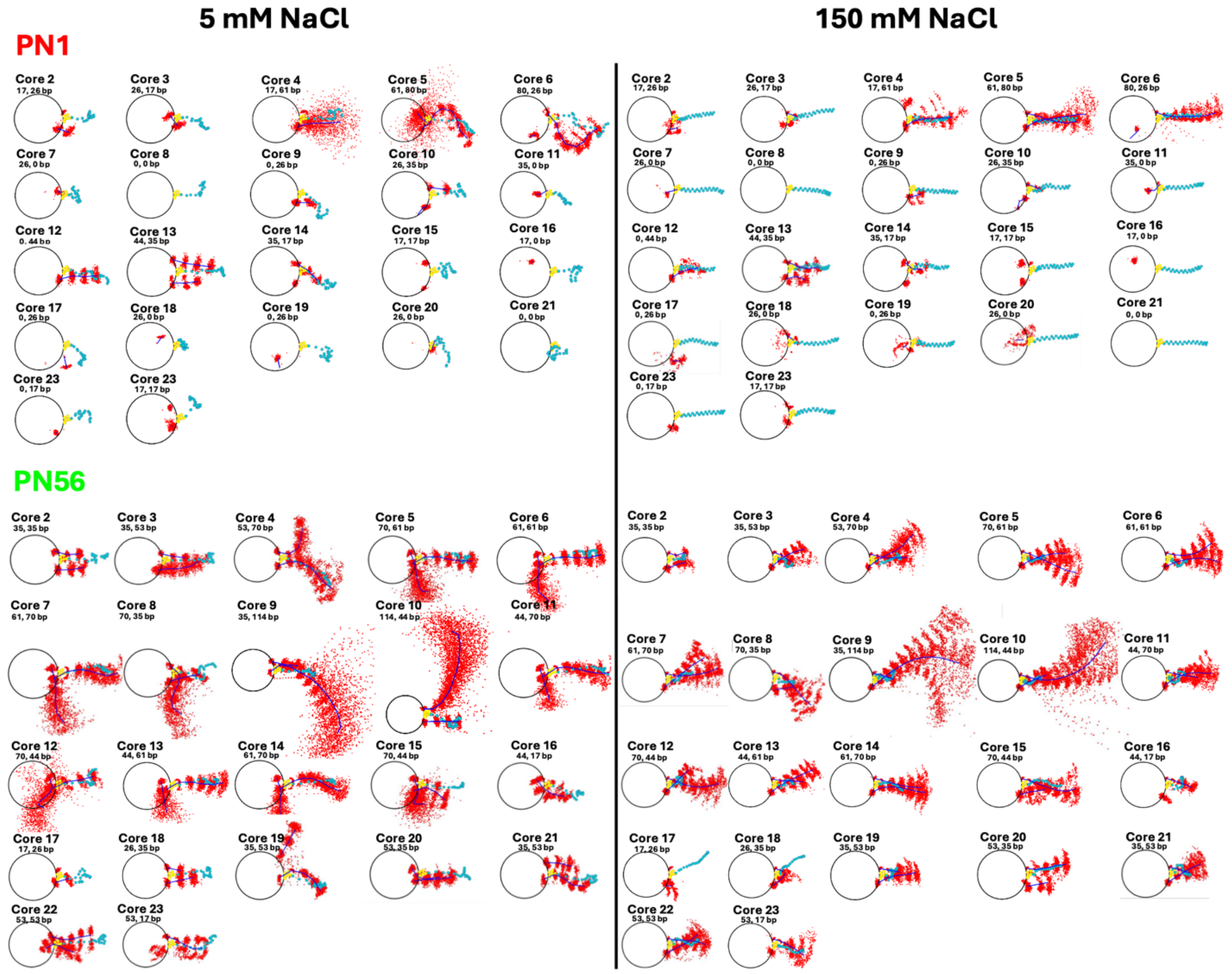

Suppl. Figure S5 (related to Fig. 7). Formation of nucleosome linker stems strongly depends on the linker DNA length.

For the PN1 and PN56 systems we show “linker DNA fan plots” for each core calculated over a single trajectory of length 40 million Monte Carlo steps at different salt conditions: 5 and 150 mM NaCl. Red dots represent the cumulative distribution of the linker DNAs during the single trajectory. Blue lines represent the average position of each linker DNA. Linker histone globular domain (6 beads in our coarse grained model) is shown in yellow, and the C-terminal domain (22 beads in our coarse grained model) is shown in cyan.

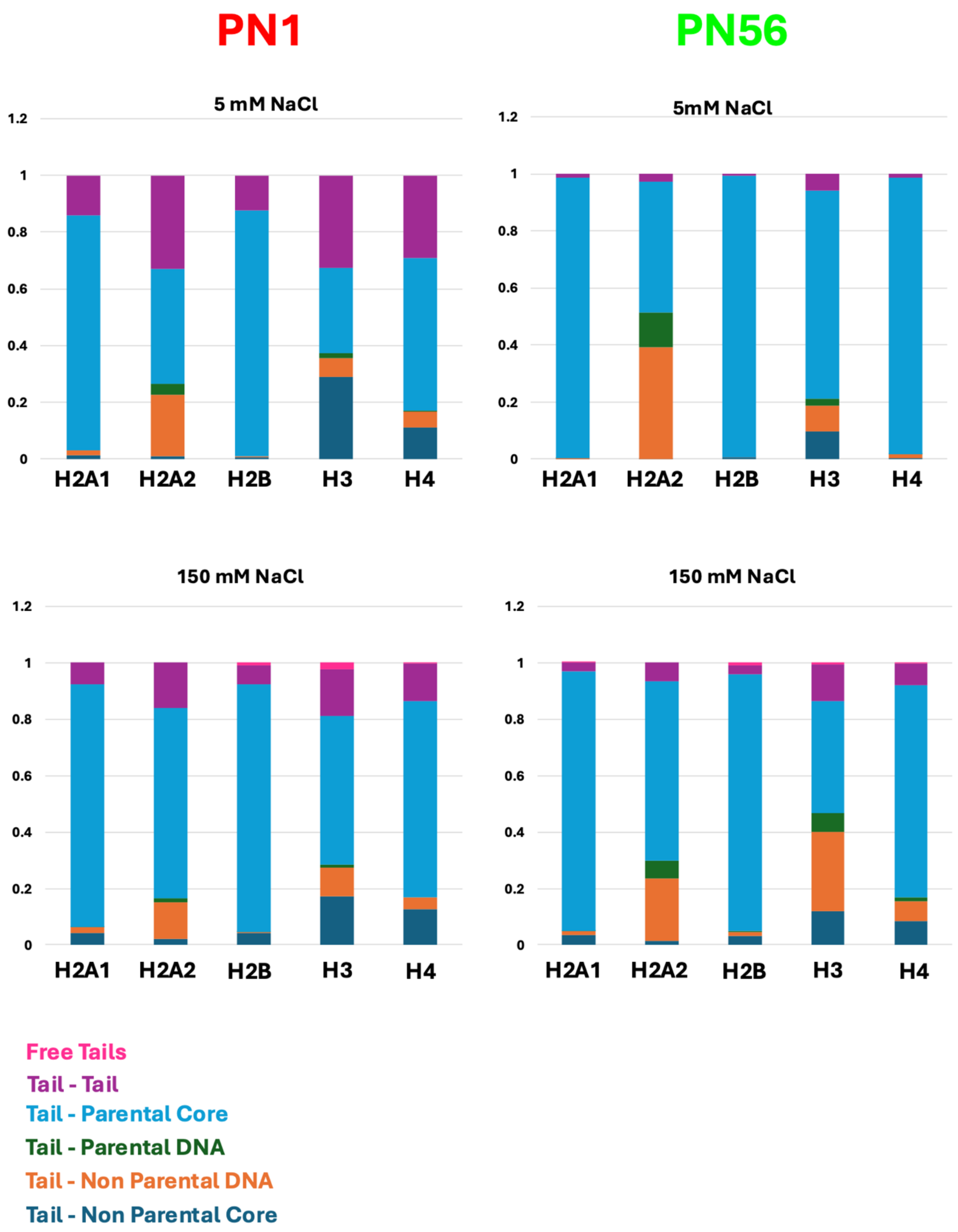

Suppl. Figure S6 (related to Fig. 7). Non-parental and parental histone N-tail interactions mediating nucleosomal contacts and chromatin folding.
For the PN1 and PN56 systems at salt conditions 5 and 150 mM NaCl we show in normalized stacked bar plots the frequency of the interactions that each tail: H2A1 (N-terminal), H2A2 (C-terminal), H2B (N-terminal)), H3 (N-terminal), and H4 (N-terminal) establishes with another tail, parental core, parental DNA, non-parental DNA, and non-parental cores. A tail is considered in contact with another chromatin element if the distance between them is less than 2 nm.
